# Influence of Talc in Polypropylene on Total Fluorine Measurements Used as an Indicator of Per- and Polyfluoroalkyl Substances (PFAS)

**DOI:** 10.1093/jaoacint/qsae090

**Published:** 2024-11-15

**Authors:** Greg W Curtzwiler, Sarah A Applegate, Mark R Early, Katie M Updegraff, Keith L Vorst

**Affiliations:** Iowa State University, Polymer and Food Protection Consortium, Ames, IA 50011, USA; Iowa State University, Department of Food Science and Human Nutrition, Ames, IA 50014, USA; Iowa State University, Polymer and Food Protection Consortium, Ames, IA 50011, USA; Iowa State University, Department of Food Science and Human Nutrition, Ames, IA 50014, USA; Iowa State University, Polymer and Food Protection Consortium, Ames, IA 50011, USA; Iowa State University, Department of Food Science and Human Nutrition, Ames, IA 50014, USA; Iowa State University, Polymer and Food Protection Consortium, Ames, IA 50011, USA; Iowa State University, Department of Food Science and Human Nutrition, Ames, IA 50014, USA; Iowa State University, Polymer and Food Protection Consortium, Ames, IA 50011, USA; Iowa State University, Department of Food Science and Human Nutrition, Ames, IA 50014, USA

## Abstract

**Background:**

Increasing restrictions for chemicals of concern in plastic packaging materials have created an urgent need to accurately detect and quantify these chemicals. Total fluorine measurements have been utilized to screen for highly scrutinized per- and polyfluorinated substances (PFAS) in food packaging materials. Inorganic contributions to the total fluorine signal can result in false positive signals exceeding regulatory limits.

**Objective:**

The purpose of this study is to develop a method for determining the contribution of talc inorganic filler to the total fluorine signal.

**Method:**

The influence of talc on total fluorine measurements of plastics was evaluated by compounding talc with virgin polypropylene (PP) and then measuring the total fluorine concentration using oxidative pyrohydrolytic combustion ion chromatography. This study provides a framework to predict the contribution of talc in plastic samples to the total fluorine signal.

**Results:**

A near-infrared spectroscopy (NIR) method was developed by employing the full width half height (FWHH) of the interstitial fluorine characteristic band of talc. The FWHH signal of the processed puck specimens was determined to be linearly increase with the measured total fluorine difference as a function of talc concentration (R^2^ = 0.9619).

**Conclusions:**

This study developed a method to predict the contribution of talc fillers to the total fluorine signal of plastic samples. This method is critical for accurately determining the regulatory compliance of talc-filled plastic samples for PFAS using total fluorine.

**Highlights:**

Total fluorine is a common regulatory compliance technique as an indicator of PFAS. Talc is a common plastic filler that contains fluorine as a contaminant. The fluorine in talc contributes to the total fluorine signal, which can falsely elevate the total fluorine signal, potentially resulting in the lack of regulatory compliance. The developed method serves as a framework of how to identify the fluorine contribution of inorganic fillers in plastics.

Per- and polyfluorinated substances (PFAS) are a group of greater than 15 000 chemical compounds identified as chemicals of concern for food packaging materials and persistent organic pollutants in the environment (1–3). According to the Centers for Disease Control and Prevention (CDC), PFAS compounds are used in a variety of products, including food packaging and cooking surfaces (2). PFAS are a major concern for regulatory authorities because they are considered “forever chemicals” that do not break down easily under standard environmental conditions. PFAS compounds have the ability to permeate through soil and travel into groundwater, contaminating drinking water through rivers, lakes, and streams. PFAS compounds also have the ability to bioaccumulate in wildlife and aquatic life (2). Since PFAS are not easily broken down, they can remain present in the food packaging supply chain from incorporating recycled materials containing previously approved oil and grease-resistant (OGRs) coatings and polymer processing aids (PPAs). PFAS has the potential to migrate to food, which can be consumed by consumers or enter into the environment after disposal (2).

As PFAS are being phased out of packaging (4), accurate monitoring and proper detection methods must be established to identify their presence in food packaging materials. PFAS may be present in food packaging due to contamination or impurity from water used in the manufacturing process originating in the environmental surroundings (i.e., lakes, rivers, streams) (5). Total fluorine analysis can be used as an indicator of PFAS in food packaging products, but it cannot distinguish between these PFAS compounds or other fluorine substances (5). This is a key area for research to distinguish when testing for total fluorine with respect to regulatory thresholds. PFAS compounds in food packaging materials and the fluoride ion that is present from other inorganic sources such as minerals used as bulking/filling agents (i.e., talc) also contain fluoride when detected through methods such as total fluorine analysis.

Talc is commonly used as an inorganic filler in plastic food packaging materials as well as biopolymer materials (6). Talc is a mineral with layers of magnesium silicate, which are held together by weak van der Waals forces. The chemical composition of talc (Mg_3_Si_4_O_10_(OH)_2_) has been evaluated throughout the literature as hydrophobic, and it is widely used as a nucleating aid for various polymer matrixes. The soft nature of talc allows for dispersion of the material throughout polymer matrixes, which substantiates its use as a filler commonly used in plastic packaging (7–10). A current challenge of using talc as a filler material is the fluoride contamination interference and the contribution to the total fluorine signal, which is commonly used as an indicator of PFAS presence.

The impact of talc content in plastic samples on the total fluorine content was measured using oxidative pyrohydrolytic combustion ion chromatography. A previous study conducted by Ignacio et al. evaluated total fluorine with oxygen combustion followed by ion selective electrode (11). This study utilized oxidative pyrohydrolytic combustion ion chromatography to determine the contribution of talc to total fluorine measurements and to develop a predictive model using near-infrared spectroscopy (NIR). This enables subtraction of the talc contribution of the total fluorine signal to achieve organic fluorine content of plastic samples containing talc. The ability to accurately differentiate organic and inorganic fluorine in talc-filled plastic fills much-needed data gaps when determining the impact of talc on fluorine measurements. The presence of fluoride in talc as a filler in plastic would increase the overall concentration of fluoride in the plastic sample. The methods described within allow the correlation of the amount of fluoride in the sample to be quickly evaluated to determine the regulatory compliance of packaging material. The previously obtained linear correlation between fluorine content in talc and full width half height (FWHH) is a key concept for this experimentation which supports the findings from Tamburini et al. (12).

Tamburini et al. utilized NIR to predict the fluorine content in polylactide (PLA)-talc blends and found a linear correlation between the FWHH and fluorine content (12). This linear correlation of half height width of the fluoride characteristic band of Mg_3_OH centered at wavenumber 7185 cm^−1^, as indicated by Petit et al., was used as a reference for analysis (13). It is hypothesized that NIR spectral analysis can be used to predict the fluoride contribution of talc in total fluorine analysis. The FWHH measurement obtained from nondestructive method (NIR) can be efficiently found through spectral analysis and curve fitting. The impact from the findings of this study enables quick prediction for fluoride contribution of talc to total fluorine analysis of talc-filled plastics.

## Experimental

### Manufacturing Puck Talc-PP Loaded Samples With a Universal Film Maker

Virgin polypropylene (vPP) pellets (Continental Express—C/O PPR, Wood Dale, IL, USA; MFI ∼20 g/10 min) were ground with an IKA A11 Basic Analytical blade mill (IKA Works, Inc., Wilmington, NC, USA) for 1 min increments until the particle size was visually homogenized under cryogenic conditions. Size-reduced polypropylene (PP) was physically mixed with laboratory grade talc (T4-500; Thermo Fisher Scientific, Ottawa, Ontario) at eight different concentrations and then melt mixed using a SJ25 mini single screw extruder (RobotDigg, Techtongda, China). The extruder temperature profile was 50–210°C. The extrudate strand was cryogenically milled and then re-extruded to maximize talc dispersion in the PP. Samples were then dried in an oven set to 50°C for 11 days.

**Figure 1.**
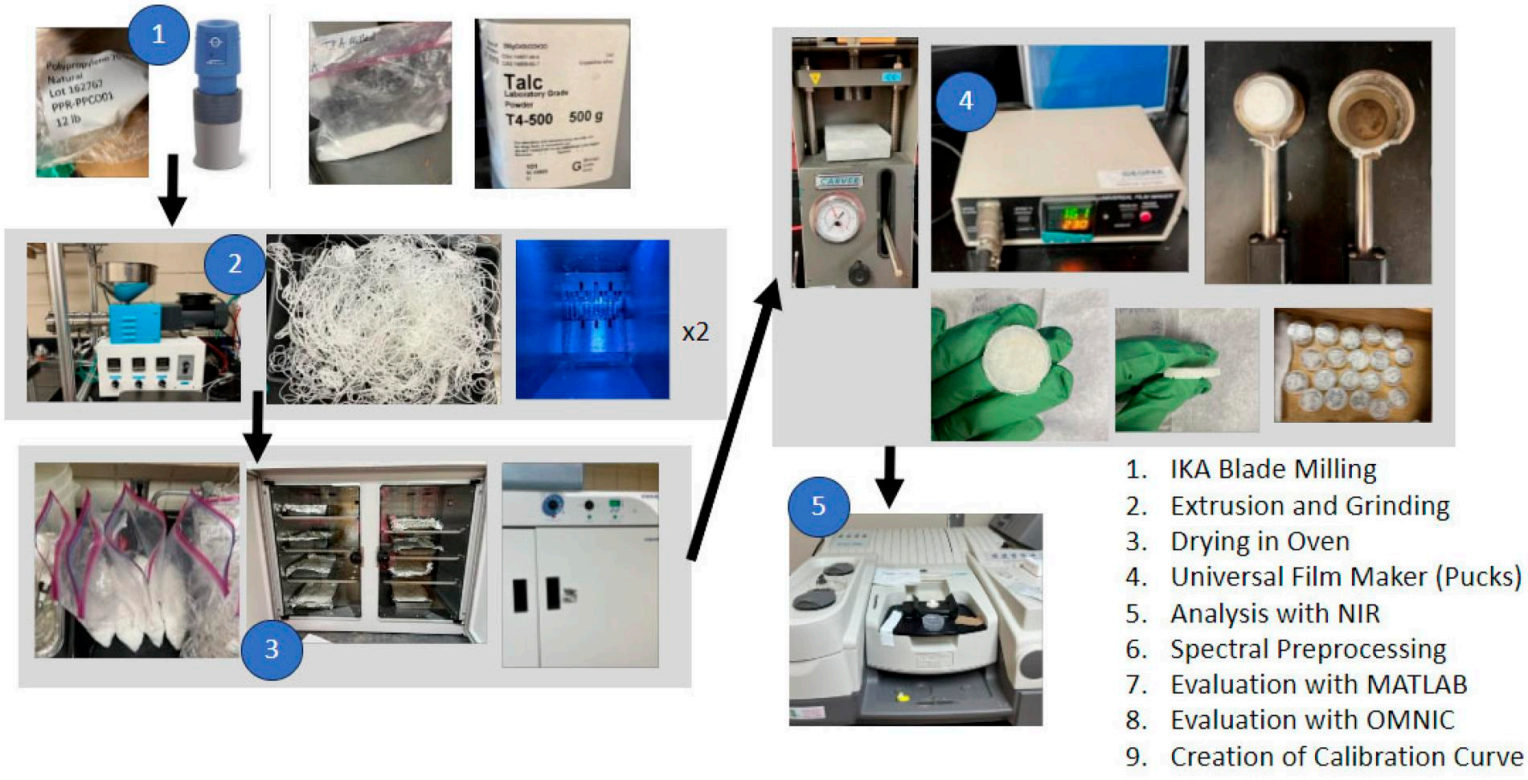
Process flow diagram for blended talc samples used to create calibration curve for FWHH versus talc loading percentage.

The milled samples were pressed with a Carver Press (model 4350CE.L; Carver, Inc. Wabash, IN, USA) and Universal Film Maker (Thermo Fisher Scientific, Madison, WI, USA) with a steel shim insert and prepped with mold release spray (Sprayon^®^ MR302, Clevland, OH, USA) to mold the samples into pucks. Near-infrared spectra between 10 000 and 6000 wavenumber (cm^−1^) were collected for both the milled and puck samples using a Nicolet 6700 FT-IR (Thermo Electron Corporation, Madison, WI, USA) with five measurements for each formulation. Milled samples were placed in a PP container prior to analysis. A Fluorilon™ wafer (Avian Technologies LLC, New London, NH) was placed on top of the puck samples prior to spectral analysis performed with NIR—Nicolet 6700 FT-IR. The calculation of FWHH was determined using Thermo Scientific™ OMNIC™ software and MATLAB^®^ R2023b software (MathWorks, Inc., Natick, MA, USA). The spectra of virgin PP pucks were used for spectral subtraction of based resin by milling pure vPP, extruding, grinding, extruding, and grinding followed by the formation of pucks and analyzed with NIR. Spectral subtraction in OMNIC for each formulation was performed on spectra by subtracting the pure processed PP spectrum. Spectra for each formulation were imported to MATLAB software to preprocess data using algorithms for the corresponding puck forms, and then FWHH was determined using peak resolve function of the OMNIC software. Statistical analysis was performed using JMP Pro 17 software (JMP Statistical Discovery LLC, Cary, NC, USA). Figure 1 is a process flow diagram for the production of puck samples.

vPP material was cryomilled with an IKA A11 analytical mill (IKA Works, Inc., Wilmington, NC, USA) using liquid nitrogen, 99.5%. Talc was mixed with vPP material at concentrations of 0, 0.5, 1, 2.5, 5, 10, 15, and 20 wt%. Ground material consisting of talc and PP was extruded using the single screw extruder set to 210°C. The extruder was initially purged with vPP followed by talc-filled formulations from lowest to highest talc concentration. The extrudate was individually ground using a Felfil model 750 grinder (Felfil Srl, Via Sant’Ambrogio 28, 10139 Torino). The grinder was vacuum-cleaned followed by high pressure air to remove residual material prior to processing subsequent formulations.

After all samples were ground in the small grinder, they were extruded a second time to increase talc dispersion in the PP. The extruder was purged with 100% vPP material prior to the second extrusion run to clean the extrusion system prior to reprocessing lower concentration formulations. Processing started with the lowest weight percent (0 wt%) talc and finished with the highest weight percent of talc (20 wt%). After samples were extruded for a second time, samples were size reduced using a mini-grinder and physically mixed for homogenization. The talc-PP samples were placed in an oven at 50°C to dry and were removed from the oven after 11 days to ensure the samples were dried completely prior to further analysis and processing.

Samples were compression-molded using a Universal Film Maker set to 230°C and a Carver Press. Heated samples were placed under a compressive load at 1000 psi (or 7 × 10^6^ Pa) for 15 s and then cooled to 50°C before removing from the apparatus. This process was repeated for all formulations to obtain five specimens each with a thickness of 3.4 mm (*see*  Supplemental Figure S1).

### Near-Infrared Spectroscopy (NIR) Analysis for Talc-PP Pucks

Thermo Scientific guidance referencing the European Pharmacopoeia 10.7 Chapter 2.2.48 (EDQM Council of Europe, Strasbourg, France) was referenced to evaluate the near-infrared absorbance of each specimen between 10 000 and 6000 cm^−1^. Spectra were collected using a NICOLET 6700 with SMART Near-IR Updrift and screens (Thermo Scientific, Sunnyvale, CA, USA). The baseline was corrected by utilizing the reference curve of pure PP processed spectra which was processed PP puck spectrum. The curve subtraction allowed for the PP spectra to be subtracted from PP-talc spectra in order for talc spectra to be preprocessed (*see*  Supplemental Figures S2-S4). FWHH was obtained through utilization of the OMNIC software peak resolve function (*see*  Supplemental Figure S5).

### NIR Experimental Design Powder Retsch Ball Mill Materials and Methods

**Figure 2.**
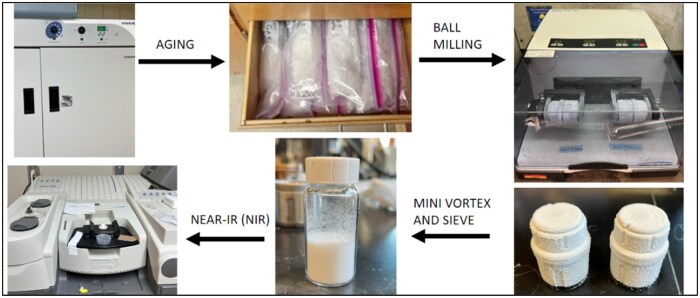
Process flow diagram for cryogenically ball-milled samples using the Retsch ball mill to obtain NIR spectra peaks for samples.

A Retsch Ball Mill Model MM 400 (Retsch GmbH Haan, Germany) was utilized to obtain cryogenically ground/milled samples for each talc loading percentage following the process flow diagram in Figure 2. Samples were immersed in liquid nitrogen 99.5% in ball mill containers for 5 min and then milled with a frequency of 30 1/s and a duration of 30 s. Each sample was cryogenically ground through at least 10 cycles before sieving through a 425 µm stainless-steel testing sieve with mesh and frame (Endecotts Ltd, London, UK). Sieved samples were collected and contained in glass scintillation vials with target heights of 20 mm. Remaining samples were placed in another scintillation vial for storage. NIR spectra of powder samples were collected using the same parameters as above. The same spectral subtraction to remove the pure PP signal was completed as with the puck sample form. Powder samples were preprocessed using MATLAB algorithms prior to determination of the FWHH calculation using OMNIC software.

### Total Fluorine via Oxidative Pyrohydrolytic Combustion Ion Chromatography

The cryomilled formulations of each formulation were independently processed through oxidative pyrohydrolytic combustion (X-PREP, Trace Elemental Instruments, Houston, TX, USA) followed by fluoride quantification using high-pressure ion chromatography (HPIC) (Dionex Corporation, Sunnyvale, CA, USA). A known mass of approximately 25 mg was loaded into the autosampler in a quartz crucible (Trace Elemental Instruments, Huston, TX, USA). The processing fluid for the absorber, ultrapure water furnace solution, and UPW wash solution was 18.2 MΩ-cm water. Quartz crucibles were cleaned prior to each combustion by running the cups through the oxidative pyrohydrolytic combustion system containing 18.2 MΩ-cm water only followed by ultrasonication. The quartz crucible cups were placed in PP sample containers filled with 18.2 MΩ-cm water and then placed in a plastic rack inside a bath ultrasonicator (Sarstedt AG & Co. KG, Numbrecht, Germany) and irradiated for 10 min. The sample cups were processed twice through the ultrasonic water bath until the water in the inner PP tube was clear. This allowed for any residual powder to be cleansed from the quartz cup after combustion of samples. The final cleaning process consisted of rinsing the quartz cups five times with 18.2 MΩ-cm water. After the cups were cleaned and rinsed with methanol, they were loaded into the autosampler carousel.

### Ion Chromatography Analysis for Fluoride in Talc-PP Combusted Samples

The degradation products after combustion were analyzed using a Dionex Integrion HPIC (Dionex Corporation, Sunnyvale, CA, USA). A Dionex AS-DV autosampler set to 35°C. A Barnstead™ GenPure™ UF (Thermo Electron LED GmbH, Langenselbold, Hungary) was used to obtain 18.2 MΩ-cm water to prepare standards and as a process fluid source for the IC. Water was passed through an eluent degasser before an eluent generator (EGC 500 K_2_CO_3_ Eluent Generator value set to 5.9 mM and EPM 500 Electrolytic pH modifier set to 1.4 mM, which equilibrates to 4.5 mM), suppressor (31 mA), and operating conditions indicated an output voltage at 3.5 and current at 31 amps for the suppressor on the Chromeleon™ console. The conductivity detector (CD) total signal was near 17 µS during operation. Fluoride ions were detected with a conductivity detector set in negative ion mode. Dissolved combustion products were loaded into autosampler vials using a 5 mL pipet and capped with filter caps (Thermo Scientific, Sunnyvale, CA, USA) prior to loading into the autosampler. The injection volume was 4 mL for the IC instrument. Chromeleon Console 7.2.10 software (Thermo Fisher Scientific, Sunnyvale, CA, USA) was used to obtain data from the IC. Sample blanks included 18.2 MΩ-cm water that was obtained from a Thermo Scientific Barnstead GenPure 18.2 MΩ-cm Ultrapure water system. Chromatographic separation was obtained by utilization of a mobile phase of potassium carbonate with a concentration of 5.90 mM and a flow rate of 1.2 mL/min. EGC 500 Carbonate Mixer 4 mm, Dionex GM-4 2 mm Gradient Mixer, Dionex EGC 500 K_2_CO_3_ Eluent Generator Cartridge, Dionex Integrion RFIC, Dionex EPM 500 Electrolytic pH Modifier, Dionex AERS 500 Carbonate 4 mm, Dionex IonPac™ As22 RFIC™ 4 × 250 mm analytical loop, and Dionex IonPac AG22 RFIC 4 × 50 mm guard were used in the IC setup (Thermo Scientific, Sunnyvale, CA, USA).

### Chemometric Analysis

MATLAB R2023b software was utilized to create algorithms for preprocessing NIR spectra prior to determining the FWHH of the characteristic peak using OMNIC software. The “sgolayfilt” MATLAB function was utilized to apply the Savitzky-Golay filter (14, 15) to the NIR spectra for puck and powder samples for each run. The Savitzky-Golay filter was used as a smoothing filter for the spectra. MATLAB algorithms were created and utilized for the study (*see*  Supplemental Appendixes B and C for puck and powder samples, respectively). After application of the Savitzky-Golay filter, a standard normal variate (SNV) (16) processing method was applied. The SNV filter was utilized as statistical transformation to minimize background spectral noise and to minimize interferences of particle size/scattering. According to Fearn, it is recommended to perform standard normal variate preprocessing/transformation after filtering with Savitzky-Golay (17). By processing spectra in this order, it allows for more robust spectral matrix mathematics in the algorithm and allows for spectra to be preprocessed in a manner for minimizing scattering prior to application of the smoothing filter. The MATLAB R2023b code/algorithm indicates the output strategies written to obtain the preprocessed spectra that was utilized to import into OMNIC Software to conduct further processing as described in the analysis of the puck samples using NIR. The preprocessed NIR data were compared with non-preprocessed NIR data in order to determine the influence of preprocessing on FWHH. Linear regression analysis was performed on all samples post-preprocessing in order to determine the linear fit equation as well as the coefficient of determination (18, 19).

### Statistical Analysis

Statistical analysis was performed using JMP Pro 17 software (JMP Statistical Discovery LLC, Cary, NC, USA). Statistical analysis was performed to evaluate statistical significance (*P*-values) for the relationship between NIR puck FWHH and fluoride ion concentrations. One-way ANOVA was conducted for samples from NIR with JMP. Linear regression analysis was conducted for powder and puck sample correlation from NIR and powder oxidative pyrohydrolytic combustion (XPREP C-IC) using Excel software to obtain coefficient of determination. One-way ANOVA was conducted for the oxidative pyrohydrolytic combustion (XPREP C-IC) results utilizing JMP.

### Quantitation

Standards for ion chromatography were prepared from a 1000 ppm sodium fluoride stock (Thermo Fisher Scientific, Chelmsford, MA, USA) and 18.2 MΩ-cm water to make final solutions 0.001, 0.01, 0.1, 1, and 10 ppm (*see*  Supplemental Figure S6). The instrumental limit of detection (ILOD) and instrumental limit of quantification (ILOQ) were determined using the standard deviation (SD) of the *y*-intercept of three independently measured calibration curves and the average slope of the calibration curves. The ILOD was calculated as 3.3 × SD/slope, and the ILOQ was 10 × SD/slope, which were determined to be 2.51 ng/mL and 7.61 ng/mL, respectively (20). The method limit of detection (MLOD = 0.66 ng/g) and quantification (MLOQ = 2.01 ng/g) were calculated using the target sample mass (25 mg).

## Results and Discussion

The influence of additives such as talc fillers in plastic packaging on the total fluorine value can result in artificially higher total fluorine concentration, which is often used to evaluate the presence of PFAS in food packaging materials. The artificially elevated total fluorine signal could falsely result in packaging material not compliant with regulatory requirements, as observed in many packaging materials tested.

### Calculation of Near-Infrared Spectra FWHH From Puck and Cryomilled Samples

The FWHH of the characteristic peak associated with interstitial fluorine of talc (7185 cm^−1^ (12, 13)) was determined by subtracting the pure processed PP spectrum from raw spectra of talc-filled samples (PP-talc from NIR), and then the spectra were preprocessed using MATLAB before using peak resolve function of the OMNIC software to determine the FWHH for each talc-PP spectrum. Peak line shape was set to Gaussian/Lorentzian analysis (18) according to European Pharmacopoeia 10.7 Chapter 2.2.48 (EDQM Council of Europe, Strasbourg, France). Since these samples contained PP as the baseline material, these measurements provided in the curves obtained from NIR were utilized to standardize the spectral subtraction using OMNIC. It was hypothesized that higher talc concentrations increased FWHH. A good linear fit between non-preprocessed FWHH and talc concentration (R^2^ = 0.8849) was observed (*see*  Supplemental Figure S7) similar to results described by Tamburini et al. (12).

Evaluation of cryomilled samples compared to puck form samples produced different results from NIR FWHH calculations. Powder form of the samples provided a weak linear correlation between FWHH and talc concentration (R^2^ = 0.0023) (*see*  Supplemental Figure S8), suggesting that forming into a puck form is critical for using this method.

### MATLAB Preprocessing of NIR Data for Puck Form of Samples

**Figure 3.**
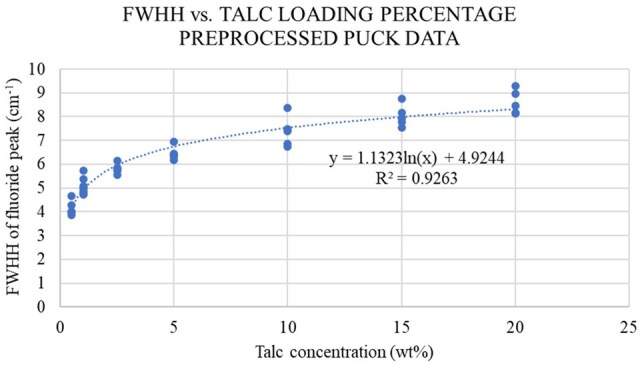
Calculated FWHH of preprocessed puck near-infrared spectra as a function of talc concentration in vPP.

Results from the two preprocessing methods (Savitzky-Golay filter (14) and standard normal variate (16)) were compared with original data obtained from NIR. A better fit (higher coefficient of determination) of FWHH as a function of talc concentration was observed by using SG and SNV preprocessing prior to determining the FWHH for puck samples using a logarithmic function (R^2^ = 0.9263; Figure 3) compared to using as-measured spectra (R^2^ = 0.8849).

MATLAB R2023b code/algorithm was developed to output the preprocessing parameters for Savitzky-Golay filtering of absorbance from powder samples (14). Standard normal variate (SNV) filtering was applied to the output from the Savitzky-Golay filtering. The same filtering strategies were applied to pure processed PP powder samples. There was a poor linear relationship between FWHH and talc loading percentage (R^2^ = 0.0204; *see*  Supplemental Figure S9) with preprocessed powder forms of sample data using MATLAB software.

### Influence of Physical Form on NIR FWHH Correlation With Talc Concentration

Statistical comparison of the physical form of talc-filled PP (cryomilled vs puck) indicated there was a stronger linear correlation for puck form between FWHH and talc concentration. It is well known that the NIR absorbance spectrum is greatly impacted by the physical state/form of the sample being analyzed (21), as observed here. Specifically, the puck sample form resulted in better linear regression fit of the data (higher coefficient of determination). Tamburini et al. compressed powder samples on a glass surface with a stainless-steel disk prior to NIR analysis (12). It is hypothesized that increased spectral quality can be achieved by compressing powder into a vial with a more uniform and flatter bottom.

Blanco et al. indicated NIR absorbance spectra is highly dependent on compactness of the sample as well as particle size (22, 23). The spectral results were similar between forms for unfilled PP. Peak height at 7185 cm^−1^ increased with increasing talc concentrations. Powder samples had an overall lower peak height for all samples measured at 7185 cm^−1^ compared to puck samples measured at 7185 cm^−1^ with and without preprocessing of the spectra using the algorithms defined above.

The results above indicate the importance of processing samples into puck form prior to analysis by NIR for determination of FWHH calculation. Results indicated a good linear fit (R^2^ > 0.8) between FWHH and talc concentration was present only when samples were processed into pucks prior to NIR analysis with or without MATLAB preprocessing. This study determined there is a weak linear fit between FWHH and talc loading for preprocessed data or non-preprocessed data for powder forms of the samples. Future research would include evaluation of a new talc sample from a different lot/batch production and cold compression of powder samples to determine variation and predictability of the correlation between FWHH and talc concentration.

### Combustion Ion Chromatography Using Oxidative Pyrohydrolytic Combustion Sample Preparation

The exact same specimen used to collect NIR spectra was used to quantify total fluorine using oxidative pyrohydrolytic combustion ion chromatography. Each formulation was measured with three unique samples as with NIR analysis.

**Figure 4.**
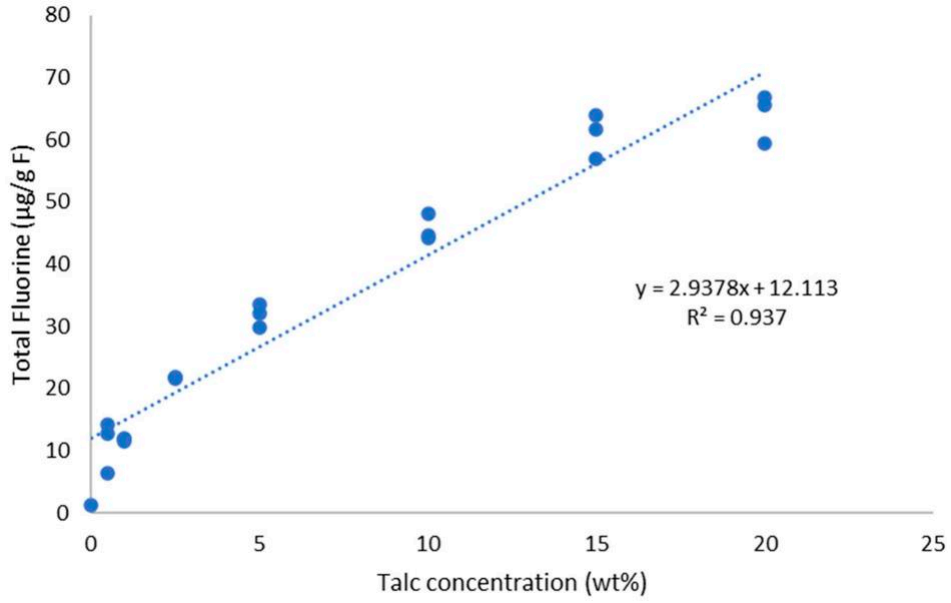
Relationship between total fluorine concentration in powder sample versus talc concentration from oxidative pyrohydrolytic C-IC. Note that the following pairs of talc concentrations were determined to have statistically the same total fluorine concentration (α = 0.05): 0.5 and 1 wt%; 15 and 20 wt%.

**Figure 5.**
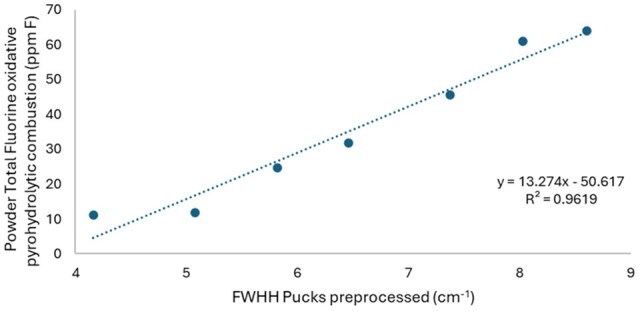
Average FWHH from the preprocessed puck samples as a function of average concentration total fluorine concentration from powder samples obtained from oxidative pyrohydrolytic combustion ion chromatography.

All values obtained for measured concentration of fluoride (ppm) for each formulation were above the method limit of quantification (MLOQ). There was a strong linear correlation (R^2^ = 0.937) between talc concentration and measured total fluorine of the powder samples (Figure 4) as anticipated. However, the total fluorine signal appears to reach a maximum around 15 wt% as the values were statistically the same as the 20 wt% talc concentration. As shown in Figure 5, there is a strong linear correlation (R^2^ = 0.9619) for average FWHH from preprocessed puck samples as a function of the concentration of fluoride from the powder samples determined by oxidative pyrohydrolytic C-IC. These data indicate the importance of processing the sample into a puck to collect the NIR spectrum but cryogrinding into powder for total fluorine processing.

The methods described herein were utilized to detect and predict the fluoride content in processed talc-loaded PP with NIR, which has applications in food packaging materials. A linear relationship was determined between the talc concentration and FWHH for puck forms of the samples and a good reproducibility for each formulation with oxidative pyrohydrolytic combustion ion chromatography. This observation agreed with previous research that the FWHH of the characteristic band related to the interstitial fluorine contamination increased with talc concentration, although the form of the sample was critical as the loose powder did not result in spectra of sufficient quality to establish the relationship, which further optimized using melt compression for a robust model. As previously described, there are a few considerations that impact the linearity between the powder NIR results and the FWHH and talc loading percentage. Therefore, melt compressing the sample into a puck appears to be necessary, although future research seeks to understand if cold compression molding (high pressure without heat) is sufficient to enable proper interaction between the infrared light and the sample. Investigating the powder density (i.e., cold compression molding) for the NIR evaluation is the subject of future research as cold compression molding is less challenging and requires less specialized equipment.

A primary outcome of this study includes the ability to use NIR to estimate the contribution of talc in the plastic sample to the total fluorine signal (i.e., the inorganic fluoride signal). This process would allow for the effect of fluoride from talc to be subtracted from plastic samples to allow for a more accurate detection of fluoride presence in the sample from PFAS and not include the fluoride from talc for regulatory consideration. Generally, higher talc concentrations in the PP yielded higher total fluorine values, as expected. However, the 15 and 20% total fluorine signals were statistically the same. This needs to be further investigated to determine if this is a limitation in the method/equipment or if higher concentrations of filler hinder fluoride release through a mixture of desorption/adsorption equilibria under the conditions utilized.

It is anticipated that other sources of talc could have different concentrations of fluoride contamination due to geographical locations of mining and refinement. This would change the linear relationship between the total fluorine response as a function of talc concentration. Such differences are anticipated to be accounted for using the NIR approach to estimate the fluoride contribution as described in this research. Furthermore, this approach can be used as a framework to estimate the fluoride contribution of other inorganic fillers such as calcium carbonate.

## Conclusions

The ability to properly discern organic fluoride from inorganic fluoride is critical for testing packaging materials for compliance with total organic fluorine regulatory thresholds. The method described herein provides an experimental framework for understanding and predicting the contribution of inorganic fluorine contamination in plastic fillers toward the total fluorine signal using oxidative pyrohydrolytic combustion ion chromatography. The FWHH of the near-infrared characteristic band related to the interstitial fluoride contamination in talc was used to establish a relationship with both the talc concentration and the resulting total fluorine concentration. This enables a rapid evaluation of a sample to determine regulatory compliance of the packaging material containing talc as a filler. Accurate detection of fluoride with combustion followed by ion chromatography and the influence of fluoride in talc was identified as a major concern with respect to accurate evaluation of PFAS compounds in plastic food packaging.

There was a strong correlation between the total fluorine concentration of powdered material using oxidative pyrohydrolytic combustion ion chromatography and talc concentration, which can be used as a predictive tool. The NIR puck correlation was determined to provide more reliable spectra for determining the FWHH compared to the powder as implemented in this research. This suggests that some standardization in sample preparation is required for this method to be used successfully, which is ongoing research.

Results of this work suggest that the impact of talc on total fluorine analysis for PFAS compounds is additive. The presence of talc may increase fluoride results for PFAS analysis to the point at which the food packaging product is no longer compliant with regulatory threshold limits. These data demonstrate the impact of talc on the overall compliance of food packaging material as total fluorine regulatory thresholds are decreasing around the world. Since the presence of talc increases the fluoride concentration of the product under analysis, PFAS concentration would ultimately be increased due to the presence of talc in the packaging sample under analysis. The presence of talc in a material may dictate regulatory compliance due to the additive effect of the fluoride content from talc during testing with combustion followed by ion chromatography and, therefore, must be accounted for through subtraction during total fluorine analysis to achieve a true assessment of total fluorine as an indicator of PFAS.

## CRediT Author Statement

Greg Curtzwiler: Conceptualization; Data curation; Funding acquisition; Methodology; Project administration; Resources; Supervision; Writing—original draft; Writing—review & editing.

Sarah Applegate: Data curation; Formal analysis; Investigation; Writing—original draft; Writing—review & editing.

Mark Early: Data curation; Methodology; Writing—review & editing.

Katherine Updegraff: Data curation; Investigation; Methodology.

Keith Vorst: Funding acquisition; Investigation; Methodology; Project administration; Supervision; Writing—review & editing.

## Supplemental Information

Supplemental information is available on the *J. AOAC Int*. website.

## Supplementary Material

qsae090_Supplementary_Data
