# Supplementary material for: Influence of Talc in Polypropylene on Total Fluorine Measurements Used as an Indicator of Per- and Polyfluoroalkyl Substances (PFAS)
Source: J AOAC Int. 2024 Nov 15;108(2):137–43. doi: 10.1093/jaoacint/qsae090 (PMC11879167; doi:10.1093/jaoacint/qsae090)
Supplement: qsae090_Supplementary_Data [file qsae090_supplementary_data.pdf]

Influence of talc in polypropylene on total fluorine measurements used as an indicator of PFAS

Greg W. Curtzwiler[1,2*], Sarah A. Applegate[1,2], Mark R. Early[1,2], Katie M. Updegraff[1,2], Keith L. Vorst[1,2]

[1]Polymer and Food Protection Consortium, Iowa State University, Ames, IA 50011, USA
[2]Department of Food Science and Human Nutrition, Iowa State University, Ames, IA 50014, USA
[*]Correspondence: gregc@iastate.edu; Tel.: +1-515-294-6146; 1555 Food Sciences Building, 536 Farmhouse Lane, Ames, IA 50014, USA

ORCID iDs:

https://orcid.org/0009-0002-3392-3541

https://orcid.org/0000-0002-0999-1037

https://orcid.org/0000-0002-5795-4791

https://orcid.org/0000-0002-2171-7161

https://orcid.org/0000-0002-4300-7937

APPENDIX A: Supplemental information for NIR and oxidative pyrohydrolytic (XPREP) combustion ion chromatography analysis

Supplemental Figures

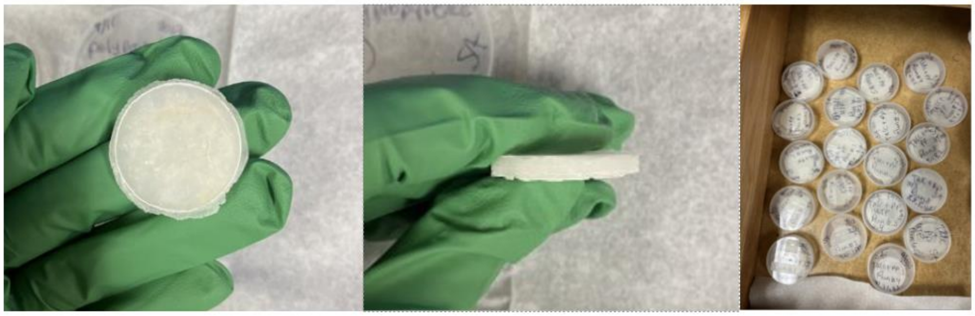

Figure S1. Puck samples manufactured via compression molding for NIR analysis.

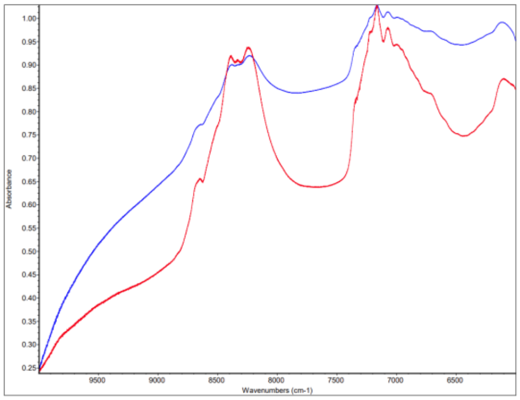

Figure S2. Absorbance spectrum for virgin polypropylene without talc in puck form (red line) and cryogenically milled polypropylene (blue line).

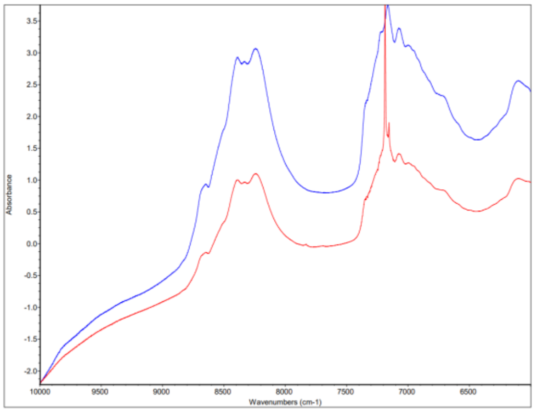

Figure S3. comparison of the measured spectrum from virgin polypropylene without talc (blue line) and with 20 wt% talc (red line). Note the sharp characteristic band of interstitial fluoride in the red line between 7500 and 7000 cm^{-1}.

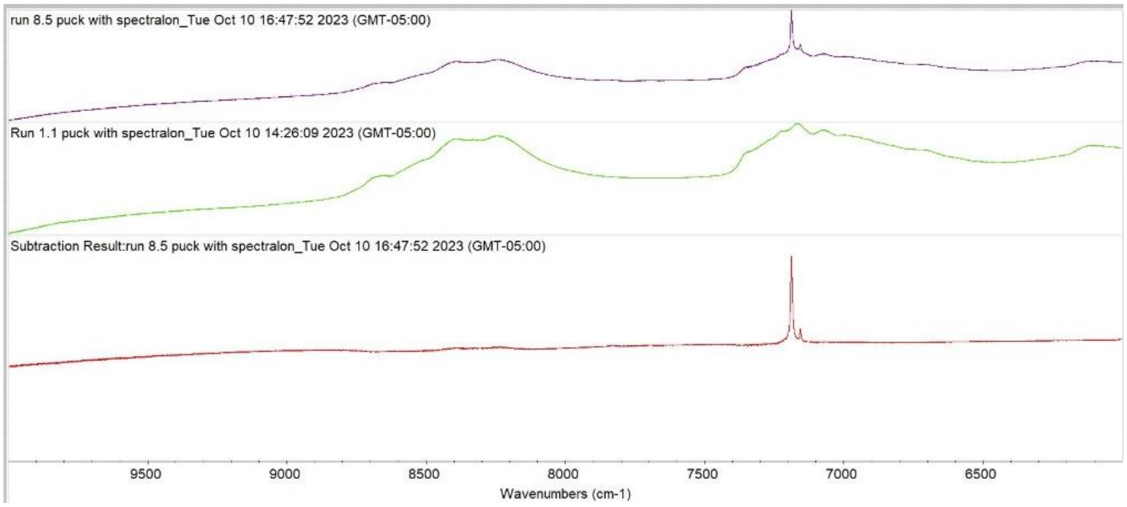

Figure S4. OMNIC™ Spectral Subtraction for the Fifth Replicate of the 20 wt% Talc-PP Puck Sample Form with Non-preprocessed Data: purple line 20 wt% talc, green line virgin polypropylene, red line subtraction result.

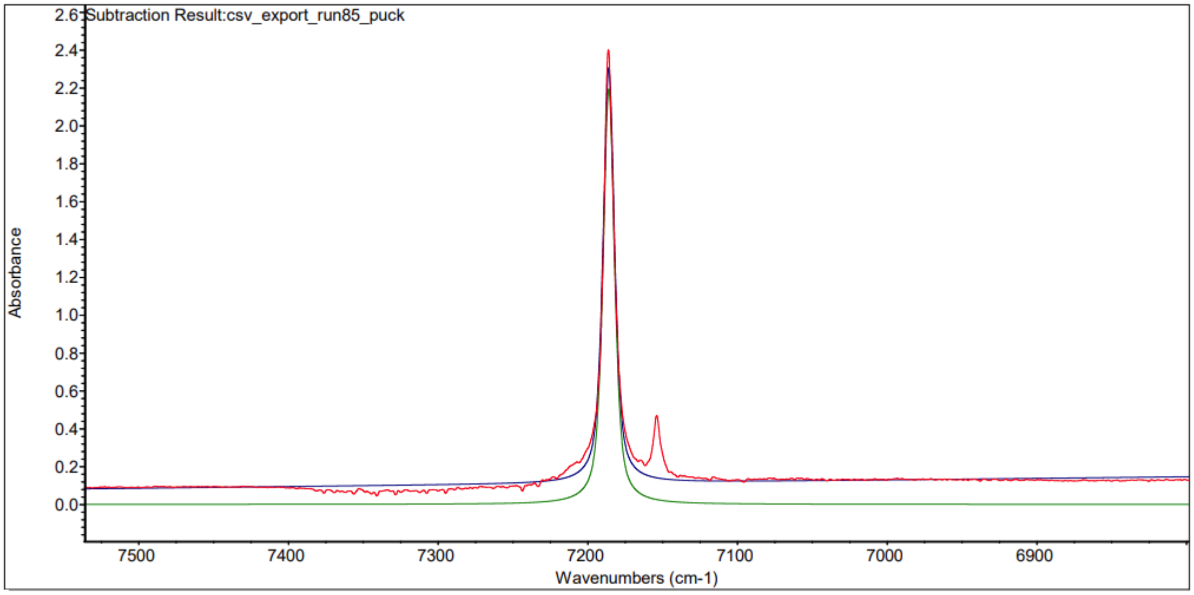

Tue Feb 06 13:21:42 2024 (GMT-06:00)
PEAKS RESOLVED:

Spectrum:	Subtraction Result:csv_export_run85_puck				
Region:	7536.360	6798.589			
Baseline:	Linear				
Peak list:					
Peak Type	Center X	Height	FWHH	Other	Area
Gaussian/Lorentzian	7185.895	2.1952	8.951	0.397	26.8474

Figure S5. Peak deconvolution analysis of 20 wt% talc-PP to calculate FWHH.

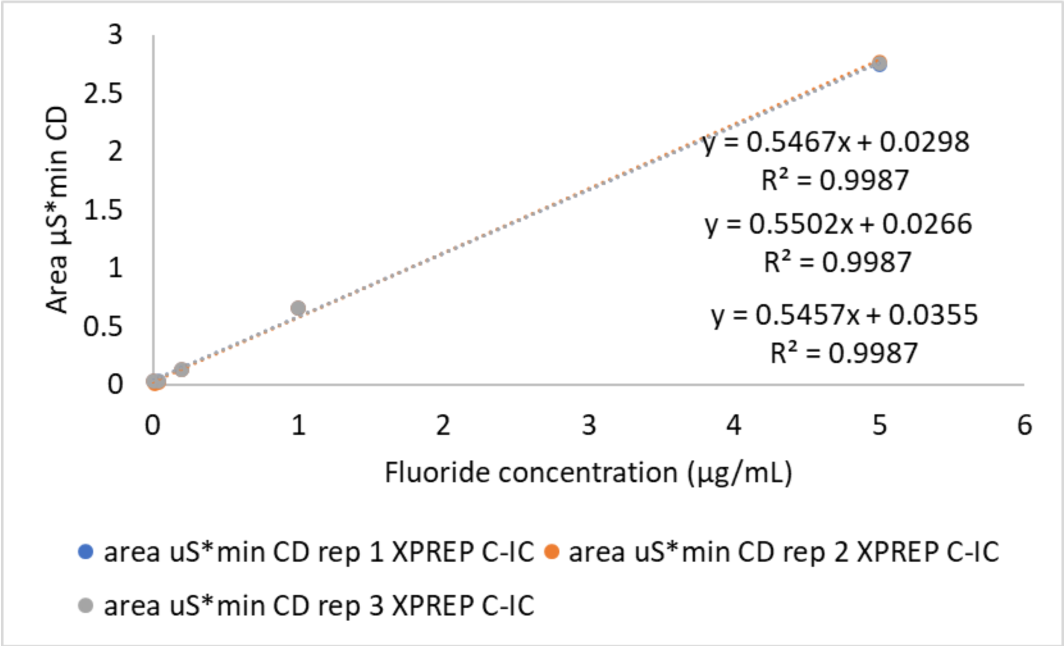

Figure S6: Calibration curves for Ion Chromatography output conductivity using a linear fit.

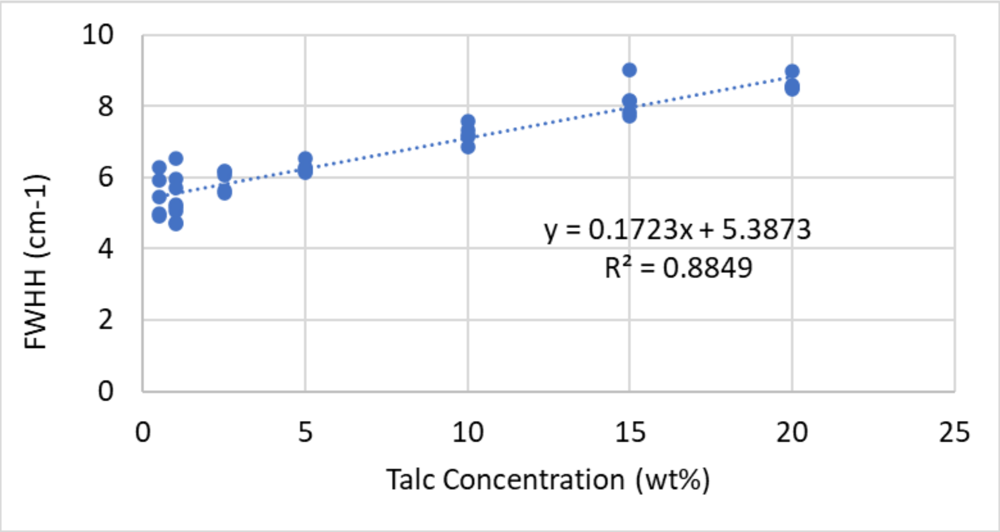

Figure S7. Calibration Curve for FWHH Versus Talc Loading Percentage for Puck Sample Forms with Non-preprocessed Data.

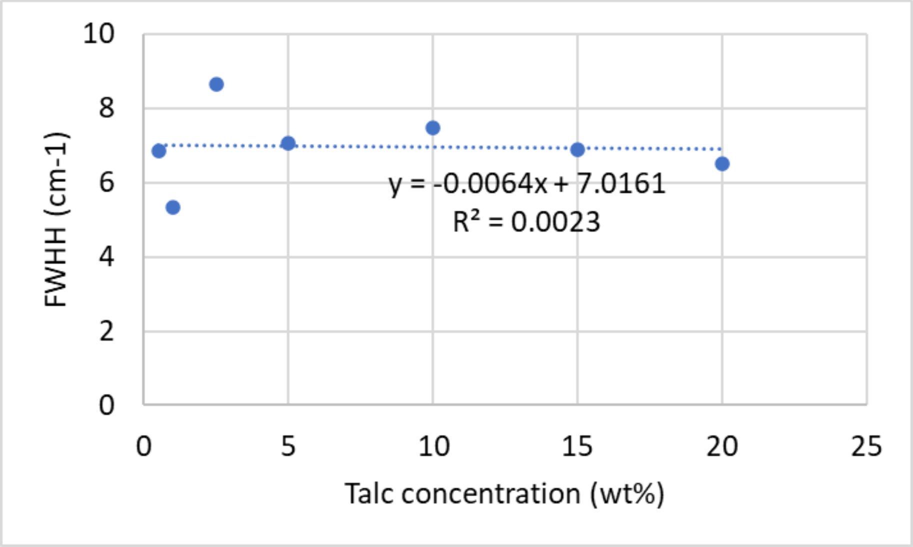

Figure S8: Full Width Half Height of Non-preprocessed Data of Powder Forms of Samples at Different Talc Loading Percentages.

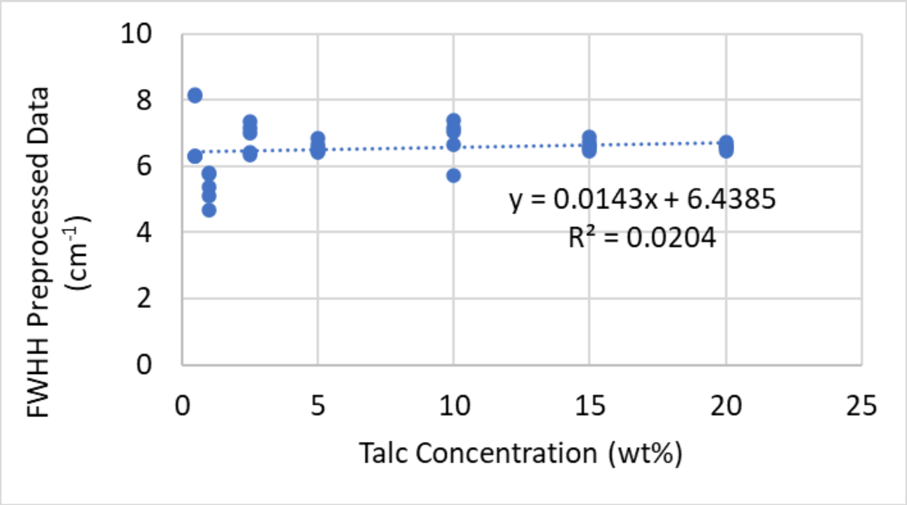

Figure S9: Correlation Between FWHH and Talc Loading Percentage with Utilization of Preprocessed Powder Data Using MATLAB® Programming for SG and SNV.

APPENDIX B: Supplemental Information for MATLAB® Code for Puck Samples

MATLAB® Code Written to Preprocess Spectral NIR Data for Puck Forms of Samples

```
%function [data] = PlotData ()

%code written by Sarah A. Applegate on 02.05.2024

function
[r_data85,r_data84,r_data83,r_data82,r_data81,r_data75,r_data74,r_data73,r_data72,r_data71,r_data65,r_data641,r_data63,r_data62,r_data61,r_data55,r_data54,r_data53,r_data52,r_data51,r_data45,r_data44,r_data43,r_data42,r_data41,r_data36,r_data35,r_data342,r_data341,r_data332,r_data331,r_data32,r_data31,r_data25,r_data11,r_wavenumber85,r_wavenumber84,r_wavenumber83,r_wavenumber82,r_wavenumber81,r_wavenumber75,r_wavenumber74,r_wavenumber73,r_wavenumber72,r_wavenumber71,r_wavenumber65,r_wavenumber641,r_wavenumber63,r_wavenumber62,r_wavenumber61,r_wavenumber55,r_wavenumber54,r_wavenumber53,r_wavenumber52,r_wavenumber51,r_wavenumber45,r_wavenumber44,r_wavenumber43,r_wavenumber42,r_wavenumber41,r_wavenumber36,r_wavenumber35,r_wavenumber342,r_wavenumber341,r_wavenumber332,r_wavenumber331,r_wavenumber32,r_wavenumber31,r_wavenumber25,r_wavenumber24,r_wavenumber23,r_wavenumber22,r_wavenumber21,r_wavenumber11,r_absorbance85,r_absorbance84,r_absorbance83,r_absorbance82,r_absorbance81,r_absorbance75,r_absorbance74,r_absorbance73,r_absorbance72,r_absorbance71,r_absorbance65,r_absorbance641,r_absorbance63,r_absorbance62,r_absorbance61,r_absorbance55,r_absorbance54,r_absorbance53,r_absorbance52,r_absorbance51,r_absorbance45,r_absorbance44,r_absorbance43,r_absorbance42,r_absorbance41,r_absorbance36,r_absorbance35,r_absorbance342,r_absorbance341,r_absorbance332,r_absorbance331,r_absorbance32,r_absorbance31,r_absorbance25,r_absorbance24,r_absorbance23,r_absorbance22,r_absorbance21,r_absorbance11] = PlotData_all_runs_pucks_v2 ()

r_data85 = xlsread('run_85.CSV');

r_data84 = xlsread('run_84.CSV');
```

```
r_data83 = xlsread('run_83.CSV');
r_data82 = xlsread('run_82.CSV');
r_data81 = xlsread('run_81.CSV');
r_data75 = xlsread('run_75.CSV');
r_data74 = xlsread('run_74.CSV');
r_data73 = xlsread('run_73.CSV');
r_data72 = xlsread('run_72.CSV');
r_data71 = xlsread('run_71.CSV');
r_data65 = xlsread('run_65.CSV');
r_data641 = xlsread('run_641.CSV');
r_data63 = xlsread('run_63.CSV');
r_data62 = xlsread('run_62.CSV');
r_data61 = xlsread('run_61.CSV');
r_data55 = xlsread('run_55.CSV');
r_data54 = xlsread('run_54.CSV');
r_data53 = xlsread('run_53.CSV');
r_data52 = xlsread('run_52.CSV');
r_data51 = xlsread('run_51.CSV');
r_data45 = xlsread('run_45.CSV');
r_data44 = xlsread('run_44.CSV');
r_data43 = xlsread('run_43.CSV');
r_data42 = xlsread('run_42.CSV');
r_data41 = xlsread('run_41.CSV');
r_data36 = xlsread('run_36.CSV');
r_data35 = xlsread('run_35.CSV');
r_data342 = xlsread('run_342.CSV');
r_data341 = xlsread('run_341.CSV');
r_data332 = xlsread('run_332.CSV');
r_data331 = xlsread('run_331.CSV');
r_data32 = xlsread('run_32.CSV');
```

```
r_data31 = xlsread('run_31.CSV');
r_data25 = xlsread('run_25.CSV');
r_data24 = xlsread('run_24.CSV');
r_data23 = xlsread('run_23.CSV');
r_data22 = xlsread('run_22.CSV');
r_data21 = xlsread('run_21.CSV');
%polypropylene processed from run 1.1
r_data11 = xlsread('run_11.CSV')
%%%Wavenumber
r_wavenumber85 = r_data85(:,1);
r_wavenumber84 = r_data84(:,1);
r_wavenumber83 = r_data83(:,1);
r_wavenumber82 = r_data82(:,1);
r_wavenumber81 = r_data81(:,1);
r_wavenumber75 = r_data75(:,1);
r_wavenumber74 = r_data74(:,1);
r_wavenumber73 = r_data73(:,1);
r_wavenumber72 = r_data72(:,1);
r_wavenumber71 = r_data71(:,1);
r_wavenumber65 = r_data65(:,1);
r_wavenumber641 = r_data641(:,1);
r_wavenumber63 = r_data63(:,1);
r_wavenumber62 = r_data62(:,1);
r_wavenumber61 = r_data61(:,1);
r_wavenumber55 = r_data55(:,1);
r_wavenumber54 = r_data54(:,1);
r_wavenumber53 = r_data53(:,1);
r_wavenumber52 = r_data52(:,1);
r_wavenumber51 = r_data51(:,1);
r_wavenumber45 = r_data45(:,1);
```

```
r_wavenumber44 = r_data44(:,1);
r_wavenumber43 = r_data43(:,1);
r_wavenumber42 = r_data42(:,1);
r_wavenumber41 = r_data41(:,1);
r_wavenumber36 = r_data36(:,1);
r_wavenumber35 = r_data35(:,1);
r_wavenumber342 = r_data342(:,1);
r_wavenumber341 = r_data341(:,1);
r_wavenumber332 = r_data332(:,1);
r_wavenumber331 = r_data331(:,1);
r_wavenumber32 = r_data32(:,1);
r_wavenumber31 = r_data31(:,1);
r_wavenumber25 = r_data25(:,1);
r_wavenumber24 = r_data24(:,1);
r_wavenumber23 = r_data23(:,1);
r_wavenumber22 = r_data22(:,1);
r_wavenumber21 = r_data21(:,1);
%polypropylene processed from run 1.1
r_wavenumber11 = r_data11(:,1);
    %wavenumber = B(:,1);
%this sets column 1 in the data matrix to wavenumber variable
r_absorbance85 = r_data85(:,2);
r_absorbance84 = r_data84(:,2);
r_absorbance83 = r_data83(:,2);
r_absorbance82 = r_data82(:,2);
r_absorbance81 = r_data81(:,2);
r_absorbance75 = r_data75(:,2);
r_absorbance74 = r_data74(:,2);
r_absorbance73 = r_data73(:,2);
r_absorbance72 = r_data72(:,2);
```

```
r_absorbance71 = r_data71(:,2);
r_absorbance65 = r_data65(:,2);
r_absorbance641 = r_data641(:,2);
r_absorbance63 = r_data63(:,2);
r_absorbance62 = r_data62(:,2);
r_absorbance61 = r_data61(:,2);
r_absorbance55 = r_data55(:,2);
r_absorbance54 = r_data54(:,2);
r_absorbance53 = r_data53(:,2);
r_absorbance52 = r_data52(:,2);
r_absorbance51 = r_data51(:,2);
r_absorbance45 = r_data45(:,2);
r_absorbance44 = r_data44(:,2);
r_absorbance43 = r_data43(:,2);
r_absorbance42 = r_data42(:,2);
r_absorbance41 = r_data41(:,2);
r_absorbance36 = r_data36(:,2);
r_absorbance35 = r_data35(:,2);
r_absorbance342 = r_data342(:,2);
r_absorbance341 = r_data341(:,2);
r_absorbance332 = r_data332(:,2);
r_absorbance331 = r_data331(:,2);
r_absorbance32 = r_data32(:,2);
r_absorbance31 = r_data31(:,2);
r_absorbance25 = r_data25(:,2);
r_absorbance24 = r_data24(:,2);
r_absorbance23 = r_data23(:,2);
r_absorbance22 = r_data22(:,2);
r_absorbance21 = r_data21(:,2);
%polypropylene processed from run 1.1
```

```
r_absorbance11 = r_data11(:,2);
    %absorbance = B(:,2);
%this sets column 2 in the data matrix to absorbance variable
subplot(6,1,1)
plot(r_wavenumber85,r_absorbance85);
hold on
plot(r_wavenumber84,r_absorbance84);
hold on
plot(r_wavenumber83,r_absorbance83);
hold on
plot(r_wavenumber82,r_absorbance82);
hold on
plot(r_wavenumber81,r_absorbance81);
hold on
plot(r_wavenumber75,r_absorbance75);
hold on
plot(r_wavenumber74,r_absorbance74);
hold on
plot(r_wavenumber73,r_absorbance73);
hold on
plot(r_wavenumber72,r_absorbance72);
hold on
plot(r_wavenumber71,r_absorbance71);
hold on
plot(r_wavenumber65,r_absorbance65);
hold on
plot(r_wavenumber641,r_absorbance641);
hold on
plot(r_wavenumber63,r_absorbance63);
hold on
```

```
plot(r_wavenumber62,r_absorbance62);
hold on
plot(r_wavenumber61,r_absorbance61);
hold on
plot(r_wavenumber55,r_absorbance55);
hold on
plot(r_wavenumber54,r_absorbance54);
hold on
plot(r_wavenumber53,r_absorbance53);
hold on
plot(r_wavenumber52,r_absorbance52);
hold on
plot(r_wavenumber51,r_absorbance51);
hold on
plot(r_wavenumber45,r_absorbance45);
hold on
plot(r_wavenumber44,r_absorbance44);
hold on
plot(r_wavenumber43,r_absorbance43);
hold on
plot(r_wavenumber42,r_absorbance42);
hold on
plot(r_wavenumber41,r_absorbance41);
hold on
plot(r_wavenumber36,r_absorbance36);
hold on
plot(r_wavenumber35,r_absorbance35);
hold on
plot(r_wavenumber342,r_absorbance342);
hold on
```

```
plot(r_wavenumber341,r_absorbance341);
hold on
plot(r_wavenumber332,r_absorbance332);
hold on
plot(r_wavenumber331,r_absorbance331);
hold on
plot(r_wavenumber32,r_absorbance32);
hold on
plot(r_wavenumber31,r_absorbance31);
hold on
plot(r_wavenumber25,r_absorbance25);
hold on
plot(r_wavenumber24,r_absorbance24);
hold on
plot(r_wavenumber23,r_absorbance23);
hold on
plot(r_wavenumber22,r_absorbance22);
hold on
plot(r_wavenumber21,r_absorbance21);
%polypropylene processed from run 1.1
grid on
%this plots the absorbance versus the wavenumber
legend('NIR spectral plot Run 8.5 Puck Form','NIR spectral plot Run 8.4 Puck Form','NIR spectral plot Run 8.3 Puck Form','NIR spectral plot Run 8.2 Puck Form','NIR spectral plot Run 8.1 Puck Form','NIR spectral plot Run 7.5 Puck Form','NIR spectral plot Run 7.4 Puck Form','NIR spectral plot Run 7.3 Puck Form','NIR spectral plot Run 7.2 Puck Form','NIR spectral plot Run 7.1 Puck Form','NIR spectral plot Run 6.5 Puck Form','NIR spectral plot Run 6.4.1 Puck Form','NIR spectral plot Run 6.3 Puck Form','NIR spectral plot Run 6.2 Puck Form','NIR spectral plot Run 6.1 Puck Form','NIR spectral plot Run 5.5 Puck Form','NIR spectral plot Run 5.4 Puck Form','NIR spectral plot Run 5.3 Puck Form','NIR spectral plot Run 5.2 Puck Form','NIR spectral plot Run 5.1 Puck Form','NIR spectral plot Run 4.5 Puck Form','NIR spectral plot Run 4.4 Puck Form','NIR spectral plot Run 4.3 Puck Form','NIR spectral plot Run 4.2 Puck Form','NIR spectral plot Run 4.1 Puck Form','NIR spectral plot Run 3.6 Puck Form','NIR spectral plot Run 3.5 Puck Form','NIR spectral plot Run 3.4.2 Puck Form','NIR spectral plot Run 3.4.1 Puck Form','NIR spectral plot Run
```

```
3.3.2 Puck Form','NIR spectral plot Run 3.3.1 Puck Form','NIR spectral plot Run 3.2 Puck Form','NIR spectral plot Run 3.1 Puck Form','NIR spectral plot Run 2.5 Puck Form','NIR spectral plot Run 2.4 Puck Form','NIR spectral plot Run 2.3 Puck Form','NIR spectral plot Run 2.2 Puck Form','NIR spectral plot Run 2.1 Puck Form')

%legend('Label 1','Label 2')

%creates a legend

xlabel('Wavenumber (cm^{-1})'), ylabel('Original Absorbance')

%creates a label for the x-axis and y-axis of the plot

title('Original Spectra Puck Forms')

%Sgolayfilt

%Savitzky-Golay Filtering

order = 1;

framelen = 15;

%wavenumber

%x data is the same for these

%absorbance

absorbance_85_sg = sgolayfilt(r_data85(:,2),order,framelen);

absorbance_84_sg = sgolayfilt(r_data84(:,2),order,framelen);

absorbance_83_sg = sgolayfilt(r_data83(:,2),order,framelen);

absorbance_82_sg = sgolayfilt(r_data82(:,2),order,framelen);

absorbance_81_sg = sgolayfilt(r_data81(:,2),order,framelen);

absorbance_75_sg = sgolayfilt(r_data75(:,2),order,framelen);

absorbance_74_sg = sgolayfilt(r_data74(:,2),order,framelen);

absorbance_73_sg = sgolayfilt(r_data73(:,2),order,framelen);

absorbance_72_sg = sgolayfilt(r_data72(:,2),order,framelen);

absorbance_71_sg = sgolayfilt(r_data71(:,2),order,framelen);

absorbance_65_sg = sgolayfilt(r_data65(:,2),order,framelen);

absorbance_641_sg = sgolayfilt(r_data641(:,2),order,framelen);

absorbance_63_sg = sgolayfilt(r_data63(:,2),order,framelen);

absorbance_62_sg = sgolayfilt(r_data62(:,2),order,framelen);

absorbance_61_sg = sgolayfilt(r_data61(:,2),order,framelen);
```

```
absorbance_55_sg = sgolayfilt(r_data55(:,2),order,framelen);
absorbance_54_sg = sgolayfilt(r_data54(:,2),order,framelen);
absorbance_53_sg = sgolayfilt(r_data53(:,2),order,framelen);
absorbance_52_sg = sgolayfilt(r_data52(:,2),order,framelen);
absorbance_51_sg = sgolayfilt(r_data51(:,2),order,framelen);
absorbance_45_sg = sgolayfilt(r_data45(:,2),order,framelen);
absorbance_44_sg = sgolayfilt(r_data44(:,2),order,framelen);
absorbance_43_sg = sgolayfilt(r_data43(:,2),order,framelen);
absorbance_42_sg = sgolayfilt(r_data42(:,2),order,framelen);
absorbance_41_sg = sgolayfilt(r_data41(:,2),order,framelen);
absorbance_36_sg = sgolayfilt(r_data36(:,2),order,framelen);
absorbance_35_sg = sgolayfilt(r_data35(:,2),order,framelen);
absorbance_342_sg = sgolayfilt(r_data342(:,2),order,framelen);
absorbance_341_sg = sgolayfilt(r_data341(:,2),order,framelen);
absorbance_332_sg = sgolayfilt(r_data332(:,2),order,framelen);
absorbance_331_sg = sgolayfilt(r_data331(:,2),order,framelen);
absorbance_32_sg = sgolayfilt(r_data32(:,2),order,framelen);
absorbance_31_sg = sgolayfilt(r_data31(:,2),order,framelen);
absorbance_25_sg = sgolayfilt(r_data25(:,2),order,framelen);
absorbance_24_sg = sgolayfilt(r_data24(:,2),order,framelen);
absorbance_23_sg = sgolayfilt(r_data23(:,2),order,framelen);
absorbance_22_sg = sgolayfilt(r_data22(:,2),order,framelen);
absorbance_21_sg = sgolayfilt(r_data21(:,2),order,framelen);
%polypropylene processed from run 1.1
absorbance_11_sg = sgolayfilt(r_data11(:,2),order,framelen);
subplot(6,1,2)
plot(r_wavenumber85,absorbance_85_sg)
hold on
plot(r_wavenumber84,absorbance_84_sg)
hold on
```

```
plot(r_wavenumber83,absorbance_83_sg)
hold on
plot(r_wavenumber82,absorbance_82_sg)
hold on
plot(r_wavenumber81,absorbance_81_sg)
hold on
plot(r_wavenumber75,absorbance_75_sg)
hold on
plot(r_wavenumber74,absorbance_74_sg)
hold on
plot(r_wavenumber73,absorbance_73_sg)
hold on
plot(r_wavenumber72,absorbance_72_sg)
hold on
plot(r_wavenumber71,absorbance_71_sg)
hold on
plot(r_wavenumber65,absorbance_65_sg)
hold on
plot(r_wavenumber641,absorbance_641_sg)
hold on
plot(r_wavenumber63,absorbance_63_sg)
hold on
plot(r_wavenumber62,absorbance_62_sg)
hold on
plot(r_wavenumber61,absorbance_61_sg)
hold on
plot(r_wavenumber55,absorbance_55_sg)
hold on
plot(r_wavenumber54,absorbance_54_sg)
hold on
```

```
plot(r_wavenumber53,absorbance_53_sg)
hold on
plot(r_wavenumber52,absorbance_52_sg)
hold on
plot(r_wavenumber51,absorbance_51_sg)
hold on
plot(r_wavenumber45,absorbance_45_sg)
hold on
plot(r_wavenumber44,absorbance_44_sg)
hold on
plot(r_wavenumber43,absorbance_43_sg)
hold on
plot(r_wavenumber42,absorbance_42_sg)
hold on
plot(r_wavenumber41,absorbance_41_sg)
hold on
plot(r_wavenumber36,absorbance_36_sg)
hold on
plot(r_wavenumber35,absorbance_35_sg)
hold on
plot(r_wavenumber342,absorbance_342_sg)
hold on
plot(r_wavenumber341,absorbance_341_sg)
hold on
plot(r_wavenumber332,absorbance_332_sg)
hold on
plot(r_wavenumber331,absorbance_331_sg)
hold on
plot(r_wavenumber32,absorbance_32_sg)
hold on
```

```
plot(r_wavenumber31,absorbance_31_sg)
hold on
plot(r_wavenumber25,absorbance_25_sg)
hold on
plot(r_wavenumber24,absorbance_24_sg)
hold on
plot(r_wavenumber23,absorbance_23_sg)
hold on
plot(r_wavenumber22,absorbance_22_sg)
hold on
plot(r_wavenumber21,absorbance_21_sg)
%polypropylene processed from run 1.1
%%polypropylene only run --> not plotted on the graph for spectral analysis
grid on
legend('Savitzky-Golay Filter applied Run 8.5 Puck Form','Savitzky-Golay Filter applied Run 8.4 Puck Form','Savitzky-Golay Filter applied Run 8.3 Puck Form','Savitzky-Golay Filter applied Run 8.2 Puck Form','Savitzky-Golay Filter applied Run 8.1 Puck Form','Savitzky-Golay Filter applied Run 7.5 Puck Form','Savitzky-Golay Filter applied Run 7.4 Puck Form','Savitzky-Golay Filter applied Run 7.3 Puck Form','Savitzky-Golay Filter Applied Run 7.2 Puck Form','Savitzky-Golay Filter Applied Run 7.1 Puck Form','Savitzky-Golay Filter Applied Run 6.5 Puck Form','Savitzky-Golay Filter Applied Run 6.4.1 Puck Form','Savitzky-Golay Filter Applied Run 6.3 Puck Form','Savitzky-Golay Filter Applied Run 6.2 Puck Form','Savitzky-Golay Filter Applied Run 6.1 Puck Form','Savitzky-Golay Filter Applied Run 5.5 Puck Form','Savitzky-Golay Filter Applied Run 5.4 Puck Form','Savitzky-Golay Filter Applied Run 5.3 Puck Form','Savitzky-Golay Filter Applied Run 5.2 Puck Form','Savitzky-Golay Filter Applied Run 5.1 Puck Form','Savitzky-Golay Filter Applied Run 4.5 Puck Form','Savitzky-Golay Filter Applied Run 4.4 Puck Form','Savitzky-Golay Filter Applied Run 4.3 Puck Form','Savitzky-Golay Filter Applied Run 4.2 Puck Form','Savitzky-Golay Filter Applied Run 4.1 Puck Form','Savitzky-Golay Filter Applied Run 3.6 Puck Form','Savitzky-Golay Filter Applied Run 3.5 Puck Form','Savitzky-Golay Filter Applied Run 3.4.2 Puck Form','Savitzky-Golay Filter Applied Run 3.4.1 Puck Form','Savitzky-Golay Filter Applied Run 3.3.2 Puck Form','Savitzky-Golay Filter Applied Run 3.3.1 Puck Form','Savitzky-Golay Filter Applied Run 3.2 Puck Form','Savitzky-Golay Filter Applied Run 3.1 Puck Form','Savitzky-Golay Filter Applied Run 2.5 Puck Form','Savitzky-Golay Filter Applied Run 2.4 Puck Form','Savitzky-Golay Filter Applied Run 2.3 Puck Form','Savitzky-Golay Filter Applied Run 2.2 Puck Form','Savitzky-Golay Filter Applied Run 2.1 Puck Form')
xlabel('Wavenumber (cm^{-1})'), ylabel('SG(Absorbance)')
title('SG Filter Spectra Puck Forms')
```

```
%SNV pretreatment
%z = (X-mu)/sigma
% X = normal random variable; mu = mean of X; sigma = std. of X
y85 = absorbance_85_sg;
y84 = absorbance_84_sg;
y83 = absorbance_83_sg;
y82 = absorbance_82_sg;
y81 = absorbance_81_sg;
y75 = absorbance_75_sg;
y74 = absorbance_74_sg;
y73 = absorbance_73_sg;
y72 = absorbance_72_sg;
y71 = absorbance_71_sg;
y65 = absorbance_65_sg;
y641 = absorbance_641_sg;
y63 = absorbance_63_sg;
y62 = absorbance_62_sg;
y61 = absorbance_61_sg;
y55 = absorbance_55_sg;
y54 = absorbance_54_sg;
y53 = absorbance_53_sg;
y52 = absorbance_52_sg;
y51 = absorbance_51_sg;
y45 = absorbance_45_sg;
y44 = absorbance_44_sg;
y43 = absorbance_43_sg;
y42 = absorbance_42_sg;
y41 = absorbance_41_sg;
y36 = absorbance_36_sg;
y35 = absorbance_35_sg;
```

```
y342 = absorbance_342_sg;
y341 = absorbance_341_sg;
y332 = absorbance_332_sg;
y331 = absorbance_331_sg;
y32 = absorbance_32_sg;
y31 = absorbance_31_sg;
y25 = absorbance_25_sg;
y24 = absorbance_24_sg;
y23 = absorbance_23_sg;
y22 = absorbance_22_sg;
y21 = absorbance_21_sg;
%polypropylene processed from run 1.1
y11 = absorbance_11_sg;
ysnv85 = (y85-mean(y85))./std(y85);
ysnv84 = (y84-mean(y84))./std(y84);
ysnv83 = (y83-mean(y83))./std(y83);
ysnv82 = (y82-mean(y82))./std(y82);
ysnv81 = (y81-mean(y81))./std(y81);
ysnv75 = (y75-mean(y75))./std(y75);
ysnv74 = (y74-mean(y74))./std(y74);
ysnv73 = (y73-mean(y73))./std(y73);
ysnv72 = (y72-mean(y72))./std(y72);
ysnv71 = (y71-mean(y71))./std(y71);
ysnv65 = (y65-mean(y65))./std(y65);
ysnv641 = (y641-mean(y641))./std(y641);
ysnv63 = (y63-mean(y63))./std(y63);
ysnv62 = (y62-mean(y62))./std(y62);
ysnv61 = (y61-mean(y61))./std(y61);
ysnv55 = (y55-mean(y55))./std(y55);
ysnv54 = (y54-mean(y54))./std(y54);
```

```
ysnv53 = (y53-mean(y53))./std(y53);
ysnv52 = (y52-mean(y52))./std(y52);
ysnv51 = (y51-mean(y51))./std(y51);
ysnv45 = (y45-mean(y45))./std(y45);
ysnv44 = (y44-mean(y44))./std(y44);
ysnv43 = (y43-mean(y43))./std(y43);
ysnv42 = (y42-mean(y42))./std(y42);
ysnv41 = (y41-mean(y41))./std(y41);
ysnv36 = (y36-mean(y36))./std(y36);
ysnv35 = (y35-mean(y35))./std(y35);
ysnv342 = (y342-mean(y342))./std(y342);
ysnv341 = (y341-mean(y341))./std(y341);
ysnv332 = (y332-mean(y332))./std(y332);
ysnv331 = (y331-mean(y331))./std(y331);
ysnv32 = (y32-mean(y32))./std(y32);
ysnv31 = (y31-mean(y31))./std(y31);
ysnv25 = (y25-mean(y25))./std(y25);
ysnv24 = (y24-mean(y24))./std(y24);
ysnv23 = (y23-mean(y23))./std(y23);
ysnv22 = (y22-mean(y22))./std(y22);
ysnv21 = (y21-mean(y21))./std(y21);
%polypropylene processed from run 1.1
ysnv11 = (y11-mean(y11))./std(y11);
%plot data
subplot(6,1,3)
plot(r_wavenumber85,ysnv85)
hold on
plot(r_wavenumber84,ysnv84)
hold on
plot(r_wavenumber83,ysnv83)
```

```
hold on
plot(r_wavenumber82,ysnv82)
hold on
plot(r_wavenumber81,ysnv81)
hold on
plot(r_wavenumber75,ysnv85)
hold on
plot(r_wavenumber74,ysnv74)
hold on
plot(r_wavenumber73,ysnv73)
hold on
plot(r_wavenumber72,ysnv72)
hold on
plot(r_wavenumber71,ysnv71)
hold on
plot(r_wavenumber65,ysnv65)
hold on
plot(r_wavenumber641,ysnv641)
hold on
plot(r_wavenumber63,ysnv63)
hold on
plot(r_wavenumber62,ysnv62)
hold on
plot(r_wavenumber61,ysnv61)
hold on
plot(r_wavenumber55,ysnv55)
hold on
plot(r_wavenumber54,ysnv54)
hold on
plot(r_wavenumber53,ysnv53)
```

```
hold on
plot(r_wavenumber52,ysnv52)
hold on
plot(r_wavenumber51,ysnv51)
hold on
plot(r_wavenumber45,ysnv45)
hold on
plot(r_wavenumber44,ysnv44)
hold on
plot(r_wavenumber43,ysnv43)
hold on
plot(r_wavenumber42,ysnv42)
hold on
plot(r_wavenumber41,ysnv41)
hold on
plot(r_wavenumber36,ysnv36)
hold on
plot(r_wavenumber35,ysnv35)
hold on
plot(r_wavenumber342,ysnv342)
hold on
plot(r_wavenumber341,ysnv341)
hold on
plot(r_wavenumber332,ysnv332)
hold on
plot(r_wavenumber331,ysnv331)
hold on
plot(r_wavenumber32,ysnv332)
hold on
plot(r_wavenumber31,ysnv31)
```

```
hold on
plot(r_wavenumber25,ysnv25)
hold on
plot(r_wavenumber24,ysnv24)
hold on
plot(r_wavenumber23,ysnv23)
hold on
plot(r_wavenumber22,ysnv22)
hold on
plot(r_wavenumber21,ysnv21)
grid on
legend('SNV Filter applied Run 8.5 Puck Form','SNV Filter applied Run 8.4 Puck Form','SNV Filter applied Run 8.3 Puck Form','SNV Filter applied Run 8.2 Puck Form','SNV Filter applied Run 8.1 Puck Form','SNV Filter applied Run 7.5 Puck Form','SNV Filter applied Run 7.4 Puck Form','SNV Filter applied Run 7.3 Puck Form','SNV Filter applied Run 7.2 Puck Form','SNV Filter applied Run 7.1 Puck Form','SNV Filter applied Run 6.5 Puck Form','SNV Filter applied Run 6.4.1 Puck Form','SNV Filter applied Run 6.3 Puck Form','SNV Filter applied Run 6.2 Puck Form','SNV Filter applied Run 6.1 Puck Form','SNV Filter applied Run 5.5 Puck Form','SNV Filter applied Run 5.4 Puck Form','SNV Filter applied Run 5.3 Puck Form','SNV Filter applied Run 5.2 Puck Form','SNV Filter applied Run 5.1 Puck Form','SNV Filter applied Run 4.5 Puck Form','SNV Filter applied Run 4.4 Puck Form','SNV Filter applied Run 4.3 Puck Form','SNV Filter applied Run 4.2 Puck Form','SNV Filter applied Run 4.1 Puck Form','SNV Filter applied Run 3.6 Puck Form','SNV Filter applied Run 3.5 Puck Form','SNV Filter applied Run 3.4.2 Puck Form','SNV Filter applied Run 3.4.1 Puck Form','SNV Filter applied Run 3.3.2 Puck Form','SNV Filter applied Run 3.3.1 Puck Form','SNV Filter applied Run 3.2 Puck Form','SNV Filter applied Run 3.1 Puck Form','SNV Filter applied Run 2.5 Puck Form','SNV Filter applied Run 2.4 Puck Form','SNV Filter applied Run 2.3 Puck Form','SNV Filter applied Run 2.2 Puck Form','SNV Filter applied Run 2.1 Puck Form')
xlabel('Wavenumber (cm^{-1})'), ylabel('SNV(SG(Absorbance))')
title('SNV Filter Spectra Puck Forms')
hold on
%polypropylene processed from run 1.1
%%polypropylene only run --> plotted on new graph for spectral analysis
subplot(6,1,4)
plot(r_wavenumber11,absorbance_11_sg)
```

```
grid on
legend('NIR spectral plot Run 1.1 Puck Form')
xlabel('Wavenumber (cm^{-1})'), ylabel('Original Absorbance')
%creates a label for the x-axis and y-axis of the plot
title('Original Spectra Puck Form')
hold on
subplot(6,1,5)
plot(r_wavenumber11,absorbance_11_sg)
grid on
%legend('NIR spectral plot Run 1.1 Puck Form','Savitzky-Golay Filter applied for Run 1.1 Puck Form')
legend('Savitzky-Golay Filter applied Run 1.1 Puck Form')
xlabel('Wavenumber (cm^{-1})'), ylabel('SG(Absorbance)')
title('SG Filter Spectra Puck Run 1.1 Puck Form')
subplot(6,1,6)
plot(r_wavenumber11,ysnv11)
grid on
legend('SNV Filter applied Run 1.1 Puck Form')
xlabel('Wavenumber (cm^{-1})'), ylabel('SNV(SG(Absorbance))')
title('SNV Filter Spectra Run 1.1 Puck Form')
%create excel file of output pretreated data for all puck sample runs
output85 = [r_wavenumber85 ysnv85];
xlswrite('export_run85_puck',output85);
output84 = [r_wavenumber84 ysnv84];
xlswrite('export_run84_puck',output84);
output83 = [r_wavenumber83 ysnv83];
xlswrite('export_run83_puck',output83);
output82 = [r_wavenumber82 ysnv82];
xlswrite('export_run82_puck',output82);
output81 = [r_wavenumber81 ysnv81];
```

```
xlswrite('export_run81_puck',output81);
output75 = [r_wavenumber75 ysnv75];
xlswrite('export_run75_puck',output75);
output74 = [r_wavenumber74 ysnv74];
xlswrite('export_run74_puck',output74);
output73 = [r_wavenumber73 ysnv73];
xlswrite('export_run73_puck',output73);
output72 = [r_wavenumber72 ysnv72];
xlswrite('export_run72_puck',output72);
output71 = [r_wavenumber71 ysnv71];
xlswrite('export_run71_puck',output71);
output65 = [r_wavenumber65 ysnv65];
xlswrite('export_run65_puck',output65);
output641 = [r_wavenumber641 ysnv641];
xlswrite('export_run641_puck',output641);
output63 = [r_wavenumber63 ysnv63];
xlswrite('export_run63_puck',output63);
output62 = [r_wavenumber62 ysnv62];
xlswrite('export_run62_puck',output62);
output61 = [r_wavenumber61 ysnv61];
xlswrite('export_run61_puck',output61);
output55 = [r_wavenumber55 ysnv55];
xlswrite('export_run55_puck',output55);
output54 = [r_wavenumber54 ysnv54];
xlswrite('export_run54_puck',output54);
output53 = [r_wavenumber53 ysnv53];
xlswrite('export_run53_puck',output53);
output52 = [r_wavenumber52 ysnv52];
xlswrite('export_run52_puck',output52);
output51 = [r_wavenumber51 ysnv51];
```

```
xlswrite('export_run51_puck',output51);
output45 = [r_wavenumber45 ysnv45];
xlswrite('export_run45_puck',output45);
output44 = [r_wavenumber44 ysnv44];
xlswrite('export_run44_puck',output44);
output43 = [r_wavenumber43 ysnv43];
xlswrite('export_run43_puck',output43);
output42 = [r_wavenumber42 ysnv42];
xlswrite('export_run42_puck',output42);
output41 = [r_wavenumber41 ysnv41];
xlswrite('export_run41_puck',output41);
output36 = [r_wavenumber36 ysnv36];
xlswrite('export_run36_puck',output36);
output35 = [r_wavenumber35 ysnv35];
xlswrite('export_run35_puck',output35);
output342 = [r_wavenumber342 ysnv342];
xlswrite('export_run342_puck',output342);
output341 = [r_wavenumber341 ysnv341];
xlswrite('export_run341_puck',output341);
output332 = [r_wavenumber332 ysnv332];
xlswrite('export_run332_puck',output332);
output331 = [r_wavenumber331 ysnv331];
xlswrite('export_run331_puck',output331);
output32 = [r_wavenumber32 ysnv32];
xlswrite('export_run32_puck',output32);
output31 = [r_wavenumber31 ysnv31];
xlswrite('export_run31_puck',output31);
output25 = [r_wavenumber25 ysnv25];
xlswrite('export_run25_puck',output25);
output24 = [r_wavenumber25 ysnv25];
```

```
xlswrite('export_run24_puck',output24);
output23 = [r_wavenumber25 ysnv25];
xlswrite('export_run23_puck',output23);
output22 = [r_wavenumber25 ysnv25];
xlswrite('export_run22_puck',output22);
output21 = [r_wavenumber25 ysnv25];
xlswrite('export_run21_puck',output21);
%polypropylene processed from run 1.1
output11 = [r_wavenumber11 ysnv11];
xlswrite('export_run11_puck',output11);

%create CSV file of output pretreated data for all puck sample runs
output85 = [r_wavenumber85 ysnv85];
csvwrite('csv_export_run85_puck',output85);
output84 = [r_wavenumber84 ysnv84];
csvwrite('csv_export_run84_puck',output84);
output83 = [r_wavenumber83 ysnv83];
csvwrite('csv_export_run83_puck',output83);
output82 = [r_wavenumber82 ysnv82];
csvwrite('csv_export_run82_puck',output82);
output81 = [r_wavenumber81 ysnv81];
csvwrite('csv_export_run81_puck',output81);
output75 = [r_wavenumber75 ysnv75];
csvwrite('csv_export_run75_puck',output75);
output74 = [r_wavenumber74 ysnv74];
csvwrite('csv_export_run74_puck',output74);
output73 = [r_wavenumber73 ysnv73];
csvwrite('csv_export_run73_puck',output73);
output72 = [r_wavenumber72 ysnv72];
csvwrite('csv_export_run72_puck',output72);
```

```
output71 = [r_wavenumber71 ysnv71];
csvwrite('csv_export_run71_puck',output71);
output65 = [r_wavenumber65 ysnv65];
csvwrite('csv_export_run65_puck',output65);
output641 = [r_wavenumber641 ysnv641];
csvwrite('csv_export_run641_puck',output641);
output63 = [r_wavenumber63 ysnv63];
csvwrite('csv_export_run63_puck',output63);
output62 = [r_wavenumber62 ysnv62];
csvwrite('csv_export_run62_puck',output62);
output61 = [r_wavenumber61 ysnv61];
csvwrite('csv_export_run61_puck',output61);
output55 = [r_wavenumber55 ysnv55];
csvwrite('csv_export_run55_puck',output55);
output54 = [r_wavenumber54 ysnv54];
csvwrite('csv_export_run54_puck',output54);
output53 = [r_wavenumber53 ysnv53];
csvwrite('csv_export_run53_puck',output53);
output52 = [r_wavenumber52 ysnv52];
csvwrite('csv_export_run52_puck',output52);
output51 = [r_wavenumber51 ysnv51];
csvwrite('csv_export_run51_puck',output51);
output45 = [r_wavenumber45 ysnv45];
csvwrite('csv_export_run45_puck',output45);
output44 = [r_wavenumber44 ysnv44];
csvwrite('csv_export_run44_puck',output44);
output43 = [r_wavenumber43 ysnv43];
csvwrite('csv_export_run43_puck',output43);
output42 = [r_wavenumber42 ysnv42];
csvwrite('csv_export_run42_puck',output42);
```

```
output41 = [r_wavenumber41 ysnv41];
csvwrite('csv_export_run41_puck',output41);
output36 = [r_wavenumber36 ysnv36];
csvwrite('csv_export_run36_puck',output36);
output35 = [r_wavenumber35 ysnv35];
csvwrite('csv_export_run35_puck',output35);
output342 = [r_wavenumber342 ysnv342];
csvwrite('csv_export_run342_puck',output342);
output341 = [r_wavenumber341 ysnv341];
csvwrite('csv_export_run341_puck',output341);
output332 = [r_wavenumber332 ysnv332];
csvwrite('csv_export_run332_puck',output332);
output331 = [r_wavenumber331 ysnv331];
csvwrite('csv_export_run331_puck',output331);
output32 = [r_wavenumber32 ysnv32];
csvwrite('csv_export_run32_puck',output32);
output31 = [r_wavenumber31 ysnv31];
csvwrite('csv_export_run31_puck',output31);
output25 = [r_wavenumber25 ysnv25];
csvwrite('csv_export_run25_puck',output25);
output24 = [r_wavenumber25 ysnv25];
csvwrite('csv_export_run24_puck',output24);
output23 = [r_wavenumber25 ysnv25];
csvwrite('csv_export_run23_puck',output23);
output22 = [r_wavenumber25 ysnv25];
csvwrite('csv_export_run22_puck',output22);
output21 = [r_wavenumber25 ysnv25];
csvwrite('csv_export_run21_puck',output21);
%polypropylene processed from run 1.1
output11 = [r_wavenumber11 ysnv11];
```

```
csvwrite('csv_export_run11_puck',output11);
end
```

APPENDIX C: Supplemental Information for MATLAB® Code for Powder Samples

MATLAB® Code Written to Preprocess Spectral NIR Data for Powder Forms of Samples

```
%function [data] = PlotData ()

%code written by Sarah A. Applegate on 02.07.2024

function [r_data85,r_data84,r_data83,r_data82,r_data81,r_data75,r_data74,r_data73,r_data72,r_data71,r_data65,r_data64,r_data63,r_data62,r_data61,r_data55,r_data54,r_data53,r_data52,r_data51,r_data45,r_data44,r_data43,r_data42,r_data41,r_data35,r_data34,r_data33,r_data32,r_data31,r_data25,r_data11,r_wavenumber85,r_wavenumber84,r_wavenumber83,r_wavenumber82,r_wavenumber81,r_wavenumber75,r_wavenumber74,r_wavenumber73,r_wavenumber72,r_wavenumber71,r_wavenumber65,r_wavenumber64,r_wavenumber63,r_wavenumber62,r_wavenumber61,r_wavenumber55,r_wavenumber54,r_wavenumber53,r_wavenumber52,r_wavenumber51,r_wavenumber45,r_wavenumber44,r_wavenumber43,r_wavenumber42,r_wavenumber41,r_wavenumber35,r_wavenumber34,r_wavenumber33,r_wavenumber32,r_wavenumber31,r_wavenumber25,r_wavenumber24,r_wavenumber23,r_wavenumber22,r_wavenumber21,r_wavenumber11,r_absorbance85,r_absorbance84,r_absorbance83,r_absorbance82,r_absorbance81,r_absorbance75,r_absorbance74,r_absorbance73,r_absorbance72,r_absorbance71,r_absorbance65,r_absorbance64,r_absorbance63,r_absorbance62,r_absorbance61,r_absorbance55,r_absorbance54,r_absorbance53,r_absorbance52,r_absorbance51,r_absorbance45,r_absorbance44,r_absorbance43,r_absorbance42,r_absorbance41,r_absorbance35,r_absorbance34,r_absorbance33,r_absorbance32,r_absorbance31,r_absorbance25,r_absorbance24,r_absorbance23,r_absorbance22,r_absorbance21,r_absorbance11] = PlotData_all_runs_powder ()

r_data85 = xlsread('run_85_powder.CSV');

r_data84 = xlsread('run_84_powder.CSV');

r_data83 = xlsread('run_83_powder.CSV');

r_data82 = xlsread('run_82_powder.CSV');

r_data81 = xlsread('run_81_powder.CSV');

r_data75 = xlsread('run_75_powder.CSV');

r_data74 = xlsread('run_74_powder.CSV');

r_data73 = xlsread('run_73_powder.CSV');

r_data72 = xlsread('run_72_powder.CSV');

r_data71 = xlsread('run_71_powder.CSV');

r_data65 = xlsread('run_65_powder.CSV');

r_data64 = xlsread('run_64_powder.CSV');

r_data63 = xlsread('run_63_powder.CSV');

r_data62 = xlsread('run_62_powder.CSV');
```

```
r_data61 = xlsread('run_61_powder.CSV');
r_data55 = xlsread('run_55_powder.CSV');
r_data54 = xlsread('run_54_powder.CSV');
r_data53 = xlsread('run_53_powder.CSV');
r_data52 = xlsread('run_52_powder.CSV');
r_data51 = xlsread('run_51_powder.CSV');
r_data45 = xlsread('run_45_powder.CSV');
r_data44 = xlsread('run_44_powder.CSV');
r_data43 = xlsread('run_43_powder.CSV');
r_data42 = xlsread('run_42_powder.CSV');
r_data41 = xlsread('run_41_powder.CSV');
r_data35 = xlsread('run_35_powder.CSV');
r_data34 = xlsread('run_34_powder.CSV');
r_data33 = xlsread('run_33_powder.CSV');
r_data32 = xlsread('run_32_powder.CSV');
r_data31 = xlsread('run_31_powder.CSV');
r_data25 = xlsread('run_25_powder.CSV');
r_data24 = xlsread('run_24_powder.CSV');
r_data23 = xlsread('run_23_powder.CSV');
r_data22 = xlsread('run_22_powder.CSV');
r_data21 = xlsread('run_21_powder.CSV');
%polypropylene processed from run 1.1
r_data11 = xlsread('run_11_powder.CSV')
%%%WAVENUMBER
r_wavenumber85 = r_data85(:,1);
r_wavenumber84 = r_data84(:,1);
r_wavenumber83 = r_data83(:,1);
r_wavenumber82 = r_data82(:,1);
r_wavenumber81 = r_data81(:,1);
r_wavenumber75 = r_data75(:,1);
```

```
r_wavenumber74 = r_data74(:,1);
r_wavenumber73 = r_data73(:,1);
r_wavenumber72 = r_data72(:,1);
r_wavenumber71 = r_data71(:,1);
r_wavenumber65 = r_data65(:,1);
r_wavenumber64 = r_data64(:,1);
r_wavenumber63 = r_data63(:,1);
r_wavenumber62 = r_data62(:,1);
r_wavenumber61 = r_data61(:,1);
r_wavenumber55 = r_data55(:,1);
r_wavenumber54 = r_data54(:,1);
r_wavenumber53 = r_data53(:,1);
r_wavenumber52 = r_data52(:,1);
r_wavenumber51 = r_data51(:,1);
r_wavenumber45 = r_data45(:,1);
r_wavenumber44 = r_data44(:,1);
r_wavenumber43 = r_data43(:,1);
r_wavenumber42 = r_data42(:,1);
r_wavenumber41 = r_data41(:,1);
r_wavenumber35 = r_data35(:,1);
r_wavenumber34 = r_data34(:,1);
r_wavenumber33 = r_data33(:,1);
r_wavenumber32 = r_data32(:,1);
r_wavenumber31 = r_data31(:,1);
r_wavenumber25 = r_data25(:,1);
r_wavenumber24 = r_data24(:,1);
r_wavenumber23 = r_data23(:,1);
r_wavenumber22 = r_data22(:,1);
r_wavenumber21 = r_data21(:,1);
%polypropylene processed from run 1.1
```

```
r_wavenumber11 = r_data11(:,1);
%this sets column 1 in the data matrix to wavenumber variable
r_absorbance85 = r_data85(:,2);
r_absorbance84 = r_data84(:,2);
r_absorbance83 = r_data83(:,2);
r_absorbance82 = r_data82(:,2);
r_absorbance81 = r_data81(:,2);
r_absorbance75 = r_data75(:,2);
r_absorbance74 = r_data74(:,2);
r_absorbance73 = r_data73(:,2);
r_absorbance72 = r_data72(:,2);
r_absorbance71 = r_data71(:,2);
r_absorbance65 = r_data65(:,2);
r_absorbance64 = r_data64(:,2);
r_absorbance63 = r_data63(:,2);
r_absorbance62 = r_data62(:,2);
r_absorbance61 = r_data61(:,2);
r_absorbance55 = r_data55(:,2);
r_absorbance54 = r_data54(:,2);
r_absorbance53 = r_data53(:,2);
r_absorbance52 = r_data52(:,2);
r_absorbance51 = r_data51(:,2);
r_absorbance45 = r_data45(:,2);
r_absorbance44 = r_data44(:,2);
r_absorbance43 = r_data43(:,2);
r_absorbance42 = r_data42(:,2);
r_absorbance41 = r_data41(:,2);
r_absorbance35 = r_data35(:,2);
r_absorbance34 = r_data34(:,2);
r_absorbance33 = r_data33(:,2);
```

```
r_absorbance32 = r_data32(:,2);
r_absorbance31 = r_data31(:,2);
r_absorbance25 = r_data25(:,2);
r_absorbance24 = r_data24(:,2);
r_absorbance23 = r_data23(:,2);
r_absorbance22 = r_data22(:,2);
r_absorbance21 = r_data21(:,2);
%polypropylene processed from run 1.1
r_absorbance11 = r_data11(:,2);
%this sets column 2 in the data matrix to absorbance variable
subplot(6,1,1)
plot(r_wavenumber85,r_absorbance85);
hold on
plot(r_wavenumber84,r_absorbance84);
hold on
plot(r_wavenumber83,r_absorbance83);
hold on
plot(r_wavenumber82,r_absorbance82);
hold on
plot(r_wavenumber81,r_absorbance81);
hold on
plot(r_wavenumber75,r_absorbance75);
hold on
plot(r_wavenumber74,r_absorbance74);
hold on
plot(r_wavenumber73,r_absorbance73);
hold on
plot(r_wavenumber72,r_absorbance72);
hold on
plot(r_wavenumber71,r_absorbance71);
```

```
hold on
plot(r_wavenumber65,r_absorbance65);
hold on
plot(r_wavenumber64,r_absorbance64);
hold on
plot(r_wavenumber63,r_absorbance63);
hold on
plot(r_wavenumber62,r_absorbance62);
hold on
plot(r_wavenumber61,r_absorbance61);
hold on
plot(r_wavenumber55,r_absorbance55);
hold on
plot(r_wavenumber54,r_absorbance54);
hold on
plot(r_wavenumber53,r_absorbance53);
hold on
plot(r_wavenumber52,r_absorbance52);
hold on
plot(r_wavenumber51,r_absorbance51);
hold on
plot(r_wavenumber45,r_absorbance45);
hold on
plot(r_wavenumber44,r_absorbance44);
hold on
plot(r_wavenumber43,r_absorbance43);
hold on
plot(r_wavenumber42,r_absorbance42);
hold on
plot(r_wavenumber41,r_absorbance41);
```

```
hold on
plot(r_wavenumber35,r_absorbance35);
hold on
plot(r_wavenumber34,r_absorbance34);
hold on
plot(r_wavenumber33,r_absorbance33);
hold on
plot(r_wavenumber32,r_absorbance32);
hold on
plot(r_wavenumber31,r_absorbance31);
hold on
plot(r_wavenumber25,r_absorbance25);
hold on
plot(r_wavenumber24,r_absorbance24);
hold on
plot(r_wavenumber23,r_absorbance23);
hold on
plot(r_wavenumber22,r_absorbance22);
hold on
plot(r_wavenumber21,r_absorbance21);
%polypropylene processed from run 1.1
grid on
%this plots the absorbance versus the wavenumber
legend('NIR spectral plot Run 8.5 Powder','NIR spectral plot Run 8.4 Powder','NIR spectral plot Run 8.3 Powder','NIR spectral plot Run 8.2 Powder','NIR spectral plot Run 8.1 Powder','NIR spectral plot Run 7.5 Powder','NIR spectral plot Run 7.4 Powder','NIR spectral plot Run 7.3 Powder','NIR spectral plot Run 7.2 Powder','NIR spectral plot Run 7.1 Powder','NIR spectral plot Run 6.5 Powder','NIR spectral plot Run 6.4 Powder','NIR spectral plot Run 6.3 Powder','NIR spectral plot Run 6.2 Powder','NIR spectral plot Run 6.1 Powder','NIR spectral plot Run 5.5 Powder','NIR spectral plot Run 5.4 Powder','NIR spectral plot Run 5.3 Powder','NIR spectral plot Run 5.2 Powder','NIR spectral plot Run 5.1 Powder','NIR spectral plot Run 4.5 Powder','NIR spectral plot Run 4.4 Powder','NIR spectral plot Run 4.3 Powder','NIR spectral plot Run 4.2 Powder','NIR spectral plot Run 4.1 Powder','NIR spectral plot Run 3.5 Powder','NIR spectral plot
```

```
Run 3.4 Powder','NIR spectral plot Run 3.3 Powder','NIR spectral plot Run 3.2 Powder','NIR spectral plot Run 3.1 Powder','NIR spectral plot Run 2.5 Powder','NIR spectral plot Run 2.4 Powder','NIR spectral plot Run 2.3 Powder','NIR spectral plot Run 2.2 Powder','NIR spectral plot Run 2.1 Powder')
    %legend('Label 1','Label 2')
    %creates a legend
    xlabel('Wavenumber (cm^{-1})'), ylabel('Original Absorbance')
    %creates a label for the x-axis and y-axis of the plot
    title('Original Spectra Powder Forms')
    %adds a title

    %pre-processing of data input from OMNIC™ into MATLAB® software
    %Sgolayfilt
    %Savitzky-Golay Filtering
    order = 1;
    framelen = 15;
    %wavenumber
    %x data is the same for these
    %absorbance
    absorbance_85_sg = sgolayfilt(r_data85(:,2),order,framelen);
    absorbance_84_sg = sgolayfilt(r_data84(:,2),order,framelen);
    absorbance_83_sg = sgolayfilt(r_data83(:,2),order,framelen);
    absorbance_82_sg = sgolayfilt(r_data82(:,2),order,framelen);
    absorbance_81_sg = sgolayfilt(r_data81(:,2),order,framelen);
    absorbance_75_sg = sgolayfilt(r_data75(:,2),order,framelen);
    absorbance_74_sg = sgolayfilt(r_data74(:,2),order,framelen);
    absorbance_73_sg = sgolayfilt(r_data73(:,2),order,framelen);
    absorbance_72_sg = sgolayfilt(r_data72(:,2),order,framelen);
    absorbance_71_sg = sgolayfilt(r_data71(:,2),order,framelen);
    absorbance_65_sg = sgolayfilt(r_data65(:,2),order,framelen);
    absorbance_64_sg = sgolayfilt(r_data64(:,2),order,framelen);
```

```
absorbance_63_sg = sgolayfilt(r_data63(:,2),order,framelen);
absorbance_62_sg = sgolayfilt(r_data62(:,2),order,framelen);
absorbance_61_sg = sgolayfilt(r_data61(:,2),order,framelen);
absorbance_55_sg = sgolayfilt(r_data55(:,2),order,framelen);
absorbance_54_sg = sgolayfilt(r_data54(:,2),order,framelen);
absorbance_53_sg = sgolayfilt(r_data53(:,2),order,framelen);
absorbance_52_sg = sgolayfilt(r_data52(:,2),order,framelen);
absorbance_51_sg = sgolayfilt(r_data51(:,2),order,framelen);
absorbance_45_sg = sgolayfilt(r_data45(:,2),order,framelen);
absorbance_44_sg = sgolayfilt(r_data44(:,2),order,framelen);
absorbance_43_sg = sgolayfilt(r_data43(:,2),order,framelen);
absorbance_42_sg = sgolayfilt(r_data42(:,2),order,framelen);
absorbance_41_sg = sgolayfilt(r_data41(:,2),order,framelen);
absorbance_35_sg = sgolayfilt(r_data35(:,2),order,framelen);
absorbance_34_sg = sgolayfilt(r_data34(:,2),order,framelen);
absorbance_33_sg = sgolayfilt(r_data33(:,2),order,framelen);
absorbance_32_sg = sgolayfilt(r_data32(:,2),order,framelen);
absorbance_31_sg = sgolayfilt(r_data31(:,2),order,framelen);
absorbance_25_sg = sgolayfilt(r_data25(:,2),order,framelen);
absorbance_24_sg = sgolayfilt(r_data24(:,2),order,framelen);
absorbance_23_sg = sgolayfilt(r_data23(:,2),order,framelen);
absorbance_22_sg = sgolayfilt(r_data22(:,2),order,framelen);
absorbance_21_sg = sgolayfilt(r_data21(:,2),order,framelen);
%polypropylene processed from run 1.1
absorbance_11_sg = sgolayfilt(r_data11(:,2),order,framelen);
subplot(6,1,2)
plot(r_wavenumber85,absorbance_85_sg)
hold on
plot(r_wavenumber84,absorbance_84_sg)
hold on
```

```
plot(r_wavenumber83,absorbance_83_sg)
hold on
plot(r_wavenumber82,absorbance_82_sg)
hold on
plot(r_wavenumber81,absorbance_81_sg)
hold on
plot(r_wavenumber75,absorbance_75_sg)
hold on
plot(r_wavenumber74,absorbance_74_sg)
hold on
plot(r_wavenumber73,absorbance_73_sg)
hold on
plot(r_wavenumber72,absorbance_72_sg)
hold on
plot(r_wavenumber71,absorbance_71_sg)
hold on
plot(r_wavenumber65,absorbance_65_sg)
hold on
plot(r_wavenumber64,absorbance_64_sg)
hold on
plot(r_wavenumber63,absorbance_63_sg)
hold on
plot(r_wavenumber62,absorbance_62_sg)
hold on
plot(r_wavenumber61,absorbance_61_sg)
hold on
plot(r_wavenumber55,absorbance_55_sg)
hold on
plot(r_wavenumber54,absorbance_54_sg)
hold on
```

```
plot(r_wavenumber53,absorbance_53_sg)
hold on
plot(r_wavenumber52,absorbance_52_sg)
hold on
plot(r_wavenumber51,absorbance_51_sg)
hold on
plot(r_wavenumber45,absorbance_45_sg)
hold on
plot(r_wavenumber44,absorbance_44_sg)
hold on
plot(r_wavenumber43,absorbance_43_sg)
hold on
plot(r_wavenumber42,absorbance_42_sg)
hold on
plot(r_wavenumber41,absorbance_41_sg)
hold on
plot(r_wavenumber35,absorbance_35_sg)
hold on
plot(r_wavenumber34,absorbance_34_sg)
hold on
plot(r_wavenumber33,absorbance_33_sg)
hold on
plot(r_wavenumber32,absorbance_32_sg)
hold on
plot(r_wavenumber31,absorbance_31_sg)
hold on
plot(r_wavenumber25,absorbance_25_sg)
hold on
plot(r_wavenumber24,absorbance_24_sg)
hold on
```

```
plot(r_wavenumber23,absorbance_23_sg)
hold on
plot(r_wavenumber22,absorbance_22_sg)
hold on
plot(r_wavenumber21,absorbance_21_sg)
%polypropylene processed from run 1.1
grid on
legend('Savitzky-Golay Filter applied Run 8.5 Powder','Savitzky-Golay Filter applied Run 8.4 Powder','Savitzky-Golay Filter applied Run 8.3 Powder','Savitzky-Golay Filter applied Run 8.2 Powder','Savitzky-Golay Filter applied Run 8.1 Powder','Savitzky-Golay Filter applied Run 7.5 Powder','Savitzky-Golay Filter applied Run 7.4 Powder','Savitzky-Golay Filter applied Run 7.3 Powder','Savitzky-Golay Filter Applied Run 7.2 Powder','Savitzky-Golay Filter Applied Run 7.1 Powder','Savitzky-Golay Filter Applied Run 6.5 Powder','Savitzky-Golay Filter Applied Run 6.4 Powder','Savitzky-Golay Filter Applied Run 6.3 Powder','Savitzky-Golay Filter Applied Run 6.2 Powder','Savitzky-Golay Filter Applied Run 6.1 Powder','Savitzky-Golay Filter Applied Run 5.5 Powder','Savitzky-Golay Filter Applied Run 5.4 Powder','Savitzky-Golay Filter Applied Run 5.3 Powder','Savitzky-Golay Filter Applied Run 5.2 Powder','Savitzky-Golay Filter Applied Run 5.1 Powder','Savitzky-Golay Filter Applied Run 4.5 Powder','Savitzky-Golay Filter Applied Run 4.4 Powder','Savitzky-Golay Filter Applied Run 4.3 Powder','Savitzky-Golay Filter Applied Run 4.2 Powder','Savitzky-Golay Filter Applied Run 4.1 Powder','Savitzky-Golay Filter Applied Run 3.5 Powder','Savitzky-Golay Filter Applied Run 3.4 Powder','Savitzky-Golay Filter Applied Run 3.3 Powder','Savitzky-Golay Filter Applied Run 3.2 Powder','Savitzky-Golay Filter Applied Run 3.1 Powder','Savitzky-Golay Filter Applied Run 2.5 Powder','Savitzky-Golay Filter Applied Run 2.4 Powder','Savitzky-Golay Filter Applied Run 2.3 Powder','Savitzky-Golay Filter Applied Run 2.2 Powder','Savitzky-Golay Filter Applied Run 2.1 Powder')
xlabel('Wavenumber (cm^{-1})'), ylabel('SG(Absorbance)')
title('SG Filter Spectra Powder Forms')
%end
%SNV pretreatment
%Standard Normal Variate
%z = (X-mu)/sigma
% X = normal random variable; mu = mean of X; sigma = std. of X
y85 = absorbance_85_sg;
y84 = absorbance_84_sg;
y83 = absorbance_83_sg;
y82 = absorbance_82_sg;
```

```
y81 = absorbance_81_sg;
y75 = absorbance_75_sg;
y74 = absorbance_74_sg;
y73 = absorbance_73_sg;
y72 = absorbance_72_sg;
y71 = absorbance_71_sg;
y65 = absorbance_65_sg;
y64 = absorbance_64_sg;
y63 = absorbance_63_sg;
y62 = absorbance_62_sg;
y61 = absorbance_61_sg;
y55 = absorbance_55_sg;
y54 = absorbance_54_sg;
y53 = absorbance_53_sg;
y52 = absorbance_52_sg;
y51 = absorbance_51_sg;
y45 = absorbance_45_sg;
y44 = absorbance_44_sg;
y43 = absorbance_43_sg;
y42 = absorbance_42_sg;
y41 = absorbance_41_sg;
y35 = absorbance_35_sg;
y34 = absorbance_34_sg;
y33 = absorbance_33_sg;
y32 = absorbance_32_sg;
y31 = absorbance_31_sg;
y25 = absorbance_25_sg;
y24 = absorbance_24_sg;
y23 = absorbance_23_sg;
y22 = absorbance_22_sg;
```

```
y21 = absorbance_21_sg;
%polypropylene processed from run 1.1
y11 = absorbance_11_sg;
ysnv85 = (y85-mean(y85))./std(y85);
ysnv84 = (y84-mean(y84))./std(y84);
ysnv83 = (y83-mean(y83))./std(y83);
ysnv82 = (y82-mean(y82))./std(y82);
ysnv81 = (y81-mean(y81))./std(y81);
ysnv75 = (y75-mean(y75))./std(y75);
ysnv74 = (y74-mean(y74))./std(y74);
ysnv73 = (y73-mean(y73))./std(y73);
ysnv72 = (y72-mean(y72))./std(y72);
ysnv71 = (y71-mean(y71))./std(y71);
ysnv65 = (y65-mean(y65))./std(y65);
ysnv64 = (y64-mean(y64))./std(y64);
ysnv63 = (y63-mean(y63))./std(y63);
ysnv62 = (y62-mean(y62))./std(y62);
ysnv61 = (y61-mean(y61))./std(y61);
ysnv55 = (y55-mean(y55))./std(y55);
ysnv54 = (y54-mean(y54))./std(y54);
ysnv53 = (y53-mean(y53))./std(y53);
ysnv52 = (y52-mean(y52))./std(y52);
ysnv51 = (y51-mean(y51))./std(y51);
ysnv45 = (y45-mean(y45))./std(y45);
ysnv44 = (y44-mean(y44))./std(y44);
ysnv43 = (y43-mean(y43))./std(y43);
ysnv42 = (y42-mean(y42))./std(y42);
ysnv41 = (y41-mean(y41))./std(y41);
ysnv35 = (y35-mean(y35))./std(y35);
ysnv34 = (y34-mean(y34))./std(y34);
```

```
ysnv33 = (y33-mean(y33))./std(y33);
ysnv32 = (y32-mean(y32))./std(y32);
ysnv31 = (y31-mean(y31))./std(y31);
ysnv25 = (y25-mean(y25))./std(y25);
ysnv24 = (y24-mean(y24))./std(y24);
ysnv23 = (y23-mean(y23))./std(y23);
ysnv22 = (y22-mean(y22))./std(y22);
ysnv21 = (y21-mean(y21))./std(y21);
%polypropylene processed from run 1.1
ysnv11 = (y11-mean(y11))./std(y11);
%plot data
subplot(6,1,3)
plot(r_wavenumber85,ysnv85)
hold on
plot(r_wavenumber84,ysnv84)
hold on
plot(r_wavenumber83,ysnv83)
hold on
plot(r_wavenumber82,ysnv82)
hold on
plot(r_wavenumber81,ysnv81)
hold on
plot(r_wavenumber75,ysnv85)
hold on
plot(r_wavenumber74,ysnv74)
hold on
plot(r_wavenumber73,ysnv73)
hold on
plot(r_wavenumber72,ysnv72)
hold on
```

```
plot(r_wavenumber71,ysnv71)
hold on
plot(r_wavenumber65,ysnv65)
hold on
plot(r_wavenumber64,ysnv64)
hold on
plot(r_wavenumber63,ysnv63)
hold on
plot(r_wavenumber62,ysnv62)
hold on
plot(r_wavenumber61,ysnv61)
hold on
plot(r_wavenumber55,ysnv55)
hold on
plot(r_wavenumber54,ysnv54)
hold on
plot(r_wavenumber53,ysnv53)
hold on
plot(r_wavenumber52,ysnv52)
hold on
plot(r_wavenumber51,ysnv51)
hold on
plot(r_wavenumber45,ysnv45)
hold on
plot(r_wavenumber44,ysnv44)
hold on
plot(r_wavenumber43,ysnv43)
hold on
plot(r_wavenumber42,ysnv42)
hold on
```

```
plot(r_wavenumber41,ysnv41)
hold on
plot(r_wavenumber35,ysnv35)
hold on
plot(r_wavenumber34,ysnv34)
hold on
plot(r_wavenumber33,ysnv33)
hold on
plot(r_wavenumber32,ysnv32)
hold on
plot(r_wavenumber31,ysnv31)
hold on
plot(r_wavenumber25,ysnv25)
hold on
plot(r_wavenumber24,ysnv24)
hold on
plot(r_wavenumber23,ysnv23)
hold on
plot(r_wavenumber22,ysnv22)
hold on
plot(r_wavenumber21,ysnv21)
grid on
legend('SNV Filter applied Run 8.5 Powder','SNV Filter applied Run 8.4 Powder','SNV Filter applied Run 8.3 Powder','SNV Filter applied Run 8.2 Powder','SNV Filter applied Run 8.1 Powder','SNV Filter applied Run 7.5 Powder','SNV Filter applied Run 7.4 Powder','SNV Filter applied Run 7.3 Powder','SNV Filter applied Run 7.2 Powder','SNV Filter applied Run 7.1 Powder','SNV Filter applied Run 6.5 Powder','SNV Filter applied Run 6.4 Powder','SNV Filter applied Run 6.3 Powder','SNV Filter applied Run 6.2 Powder','SNV Filter applied Run 6.1 Powder','SNV Filter applied Run 5.5 Powder','SNV Filter applied Run 5.4 Powder','SNV Filter applied Run 5.3 Powder','SNV Filter applied Run 5.2 Powder','SNV Filter applied Run 5.1 Powder','SNV Filter applied Run 4.5 Powder','SNV Filter applied Run 4.4 Powder','SNV Filter applied Run 4.3 Powder','SNV Filter applied Run 4.2 Powder','SNV Filter applied Run 4.1 Powder','SNV Filter applied Run 3.5 Powder','SNV Filter applied Run 3.4 Powder','SNV Filter applied Run 3.3 Powder', 'SNV Filter applied Run 3.2 Powder','SNV Filter applied Run 3.1
```

```
Powder','SNV Filter applied Run 2.5 Powder','SNV Filter applied Run 2.4 Powder','SNV Filter applied Run 2.3 Powder','SNV Filter applied Run 2.2 Powder','SNV Filter applied Run 2.1 Powder')
    xlabel('Wavenumber (cm^{-1})'), ylabel('SNV(SG(Absorbance))')
    title('SNV Filter Spectra Powder Forms')
    hold on
    %polypropylene processed from run 1.1
    subplot(6,1,4)
    plot(r_wavenumber11,absorbance_11_sg)
    grid on
    legend('NIR spectral plot Run 1.1 Powder Form')
    xlabel('Wavenumber (cm^{-1})'), ylabel('Original Absorbance')
    %creates a label for the x-axis and y-axis of the plot
    title('Original Spectra Run 1.1 Powder Form')
    hold on
    subplot(6,1,5)
    plot(r_wavenumber11,absorbance_11_sg)
    grid on
    legend('Savitzky-Golay Filter applied Run 1.1 Powder Form')
    xlabel('Wavenumber (cm^{-1})'), ylabel('SG(Absorbance)')
    title('SG Filter Spectra Run 1.1 Powder Form')
    subplot(6,1,6)
    plot(r_wavenumber11,ysnv11)
    grid on
    legend('SNV Filter applied Run 1.1 Powder Form')
    xlabel('Wavenumber (cm^{-1})'), ylabel('SNV(SG(Absorbance))')
    title('SNV Filter Spectra Run 1.1 Powder Form')
    %%%
    %create excel file of output pretreated data for all puck sample runs
    output85 = [r_wavenumber85 ysnv85];
```

```
xlswrite('export_run85_powder',output85);
output84 = [r_wavenumber84 ysnv84];
xlswrite('export_run84_powder',output84);
output83 = [r_wavenumber83 ysnv83];
xlswrite('export_run83_powder',output83);
output82 = [r_wavenumber82 ysnv82];
xlswrite('export_run82_powder',output82);
output81 = [r_wavenumber81 ysnv81];
xlswrite('export_run81_powder',output81);
output75 = [r_wavenumber75 ysnv75];
xlswrite('export_run75_powder',output75);
output74 = [r_wavenumber74 ysnv74];
xlswrite('export_run74_powder',output74);
output73 = [r_wavenumber73 ysnv73];
xlswrite('export_run73_powder',output73);
output72 = [r_wavenumber72 ysnv72];
xlswrite('export_run72_powder',output72);
output71 = [r_wavenumber71 ysnv71];
xlswrite('export_run71_powder',output71);
output65 = [r_wavenumber65 ysnv65];
xlswrite('export_run65_powder',output65);
output64 = [r_wavenumber64 ysnv64];
xlswrite('export_run64_powder',output64);
output63 = [r_wavenumber63 ysnv63];
xlswrite('export_run63_powder',output63);
output62 = [r_wavenumber62 ysnv62];
xlswrite('export_run62_powder',output62);
output61 = [r_wavenumber61 ysnv61];
xlswrite('export_run61_powder',output61);
output55 = [r_wavenumber55 ysnv55];
```

```
xlswrite('export_run55_powder',output55);
output54 = [r_wavenumber54 ysnv54];
xlswrite('export_run54_powder',output54);
output53 = [r_wavenumber53 ysnv53];
xlswrite('export_run53_powder',output53);
output52 = [r_wavenumber52 ysnv52];
xlswrite('export_run52_powder',output52);
output51 = [r_wavenumber51 ysnv51];
xlswrite('export_run51_powder',output51);
output45 = [r_wavenumber45 ysnv45];
xlswrite('export_run45_powder',output45);
output44 = [r_wavenumber44 ysnv44];
xlswrite('export_run44_powder',output44);
output43 = [r_wavenumber43 ysnv43];
xlswrite('export_run43_powder',output43);
output42 = [r_wavenumber42 ysnv42];
xlswrite('export_run42_powder',output42);
output41 = [r_wavenumber41 ysnv41];
xlswrite('export_run41_powder',output41);
output35 = [r_wavenumber35 ysnv35];
xlswrite('export_run35_powder',output35);
output34 = [r_wavenumber34 ysnv34];
xlswrite('export_run34_powder',output34);
output33 = [r_wavenumber33 ysnv33];
xlswrite('export_run33_powder',output33);
output32 = [r_wavenumber32 ysnv32];
xlswrite('export_run32_powder',output32);
output31 = [r_wavenumber31 ysnv31];
xlswrite('export_run31_powder',output31);
output25 = [r_wavenumber25 ysnv25];
```

```
xlswrite('export_run25_powder',output25);
output24 = [r_wavenumber25 ysnv25];
xlswrite('export_run24_powder',output24);
output23 = [r_wavenumber25 ysnv25];
xlswrite('export_run23_powder',output23);
output22 = [r_wavenumber25 ysnv25];
xlswrite('export_run22_powder',output22);
output21 = [r_wavenumber25 ysnv25];
xlswrite('export_run21_powder',output21);
%polypropylene processed from run 1.1
output11 = [r_wavenumber11 ysnv11];
xlswrite('export_run11_powder',output11);
%create CSV file of output pretreated data for all powder sample runs
output85 = [r_wavenumber85 ysnv85];
csvwrite('csv_export_run85_powder',output85);
output84 = [r_wavenumber84 ysnv84];
csvwrite('csv_export_run84_powder',output84);
output83 = [r_wavenumber83 ysnv83];
csvwrite('csv_export_run83_powder',output83);
output82 = [r_wavenumber82 ysnv82];
csvwrite('csv_export_run82_powder',output82);
output81 = [r_wavenumber81 ysnv81];
csvwrite('csv_export_run81_powder',output81);
output75 = [r_wavenumber75 ysnv75];
csvwrite('csv_export_run75_powder',output75);
output74 = [r_wavenumber74 ysnv74];
csvwrite('csv_export_run74_powder',output74);
output73 = [r_wavenumber73 ysnv73];
csvwrite('csv_export_run73_powder',output73);
output72 = [r_wavenumber72 ysnv72];
```

```
csvwrite('csv_export_run72_powder',output72);
output71 = [r_wavenumber71 ysnv71];
csvwrite('csv_export_run71_powder',output71);
output65 = [r_wavenumber65 ysnv65];
csvwrite('csv_export_run65_powder',output65);
output64 = [r_wavenumber64 ysnv64];
csvwrite('csv_export_run64_powder',output64);
output63 = [r_wavenumber63 ysnv63];
csvwrite('csv_export_run63_powder',output63);
output62 = [r_wavenumber62 ysnv62];
csvwrite('csv_export_run62_powder',output62);
output61 = [r_wavenumber61 ysnv61];
csvwrite('csv_export_run61_powder',output61);
output55 = [r_wavenumber55 ysnv55];
csvwrite('csv_export_run55_powder',output55);
output54 = [r_wavenumber54 ysnv54];
csvwrite('csv_export_run54_powder',output54);
output53 = [r_wavenumber53 ysnv53];
csvwrite('csv_export_run53_powder',output53);
output52 = [r_wavenumber52 ysnv52];
csvwrite('csv_export_run52_powder',output52);
output51 = [r_wavenumber51 ysnv51];
csvwrite('csv_export_run51_powder',output51);
output45 = [r_wavenumber45 ysnv45];
csvwrite('csv_export_run45_powder',output45);
output44 = [r_wavenumber44 ysnv44];
csvwrite('csv_export_run44_powder',output44);
output43 = [r_wavenumber43 ysnv43];
csvwrite('csv_export_run43_powder',output43);
output42 = [r_wavenumber42 ysnv42];
```

```
csvwrite('csv_export_run42_powder',output42);
output41 = [r_wavenumber41 ysnv41];
csvwrite('csv_export_run41_powder',output41);
output35 = [r_wavenumber35 ysnv35];
csvwrite('csv_export_run35_powder',output35);
output34 = [r_wavenumber34 ysnv34];
csvwrite('csv_export_run34_powder',output34);
output33 = [r_wavenumber33 ysnv33];
csvwrite('csv_export_run33_powder',output33);
output32 = [r_wavenumber32 ysnv32];
csvwrite('csv_export_run32_powder',output32);
output31 = [r_wavenumber31 ysnv31];
csvwrite('csv_export_run31_powder',output31);
output25 = [r_wavenumber25 ysnv25];
csvwrite('csv_export_run25_powder',output25);
output24 = [r_wavenumber25 ysnv25];
csvwrite('csv_export_run24_powder',output24);
output23 = [r_wavenumber25 ysnv25];
csvwrite('csv_export_run23_powder',output23);
output22 = [r_wavenumber25 ysnv25];
csvwrite('csv_export_run22_powder',output22);
output21 = [r_wavenumber25 ysnv25];
csvwrite('csv_export_run21_powder',output21);
%polypropylene processed from run 1.1
output11 = [r_wavenumber11 ysnv11];
csvwrite('csv_export_run11_powder',output11);
end
```
